# (Benzyl­diphenyl­phosphane-1κ*P*)[μ-bis(diphenylphosphan­yl)methane-2:3κ^2^ *P*:*P*′]nona­carbonyl-1κ^3^ *C*,2κ^3^ *C*,3κ^3^ *C*-*triangulo*-triruthenium(0)

**DOI:** 10.1107/S1600536814015475

**Published:** 2014-07-11

**Authors:** Omar bin Shawkataly, Imthyaz Ahmed Khan, Siti Syaida Sirat, Mohd Mustaqim Rosli

**Affiliations:** a Chemical Sciences Programme, School of Distance Education, Universiti Sains Malaysia, 11800 USM, Penang, Malaysia; b Department of Chemistry, Gokhale Centenary College, Ankola 581 314, NK, Karnataka, India; c X-Ray Crystallography Unit, School of Physics, Universiti Sains Malaysia, 11800 USM, Penang, Malaysia

**Keywords:** crystal structure

## Abstract

The asymmetric unit of the title compound, [Ru_3_(C_19_H_17_P)(C_25_H_22_P_2_)(CO)_9_], consists of two independent mol­ecules. The bis­(di­phenyl­phosphan­yl)methane ligand bridges an Ru—Ru bond and the benzyl­diphenyl­phosphane ligand binds to the third Ru atom. The Ru—Ru bond *cis* to the benzyl­diphenyl­phosphane ligand is the longest of the three Ru—Ru bonds in both mol­ecules. In the crystal, mol­ecules are linked by C—H⋯O hydrogen bonds, forming layers parallel to the *ac* plane. C—H⋯π contacts further stabilize the crystal packing.

## Related literature   

For general background to triruthenium complexes of the structural type [Ru_3_(CO)_9_(*L*–*L*)(*L*)] (where *L*–*L* is a bidentate ligand and *L* a monodentate ligand), see: Koutsantonis *et al.* (2002 ▶); Shawkataly *et al.* (1998 ▶, 2009 ▶, 2010 ▶, 2011 ▶, 2012 ▶). For the use of Group 15 ligands in stabilizing metal clusters, see: Bruce *et al.* (1988*a* ▶,*b* ▶). For a general method of preparation of ruthenium cluster phosphane derivatives, see: Bruce *et al.* (1983 ▶). For the stability of the temperature controller used in the data collection, see: Cosier & Glazer (1986 ▶).
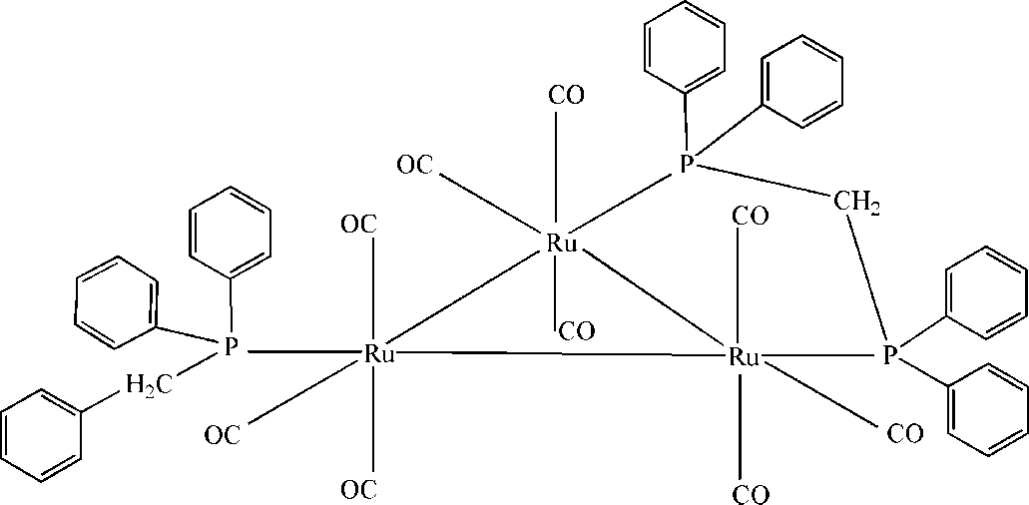


## Experimental   

### 

#### Crystal data  

[Ru_3_(C_19_H_17_P)(C_25_H_22_P_2_)(CO)_9_]
*M* *_r_* = 1215.96
Monoclinic, 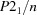

*a* = 13.4308 (2) Å
*b* = 35.3969 (6) Å
*c* = 20.6967 (3) Å
β = 97.620 (1)°
*V* = 9752.5 (3) Å^3^
*Z* = 8
Mo *K*α radiation
μ = 1.07 mm^−1^
*T* = 100 K
0.51 × 0.14 × 0.05 mm

#### Data collection  

Bruker SMART APEXII CCD area-detector diffractometer
Absorption correction: multi-scan (*SADABS*; Bruker, 2009 ▶) *T*_min_ = 0.612, *T*_max_ = 0.950
96674 measured reflections
22388 independent reflections
17595 reflections with *I* > 2σ(*I*)
*R*_int_ = 0.058

#### Refinement  

*R*[*F* ^2^ > 2σ(*F* ^2^)] = 0.043
*wR*(*F* ^2^) = 0.089
*S* = 1.06
22388 reflections
1225 parameters
H-atom parameters constrained
Δρ_max_ = 1.96 e Å^−3^
Δρ_min_ = −0.61 e Å^−3^

### 

Data collection: *APEX2* (Bruker, 2009 ▶); cell refinement: *SAINT* (Bruker, 2009 ▶); data reduction: *SAINT*; program(s) used to solve structure: *SHELXTL* (Sheldrick, 2008 ▶); program(s) used to refine structure: *SHELXTL*; molecular graphics: *SHELXTL*; software used to prepare material for publication: *SHELXTL* and *PLATON* (Spek, 2009 ▶).

## Supplementary Material

Crystal structure: contains datablock(s) I, New_Global_Publ_Block. DOI: 10.1107/S1600536814015475/sj5417sup1.cif

Structure factors: contains datablock(s) I. DOI: 10.1107/S1600536814015475/sj5417Isup2.hkl

CCDC reference: 1011638

Additional supporting information:  crystallographic information; 3D view; checkCIF report

## Figures and Tables

**Table 1:** Selected bond lengths (Å)

Ru1*A*—P1*A*	2.3353 (10)
Ru1*A*—Ru3*A*	2.8454 (4)
Ru1*A*—Ru2*A*	2.9054 (4)
Ru2*A*—P2*A*	2.3232 (10)
Ru2*A*—Ru3*A*	2.8353 (4)
Ru3*A*—P3*A*	2.3196 (10)
Ru1*B*—P1*B*	2.3406 (10)
Ru1*B*—Ru2*B*	2.8504 (4)
Ru1*B*—Ru3*B*	2.8992 (4)
Ru2*B*—P2*B*	2.3195 (10)
Ru2*B*—Ru3*B*	2.8451 (4)
Ru3*B*—P3*B*	2.3191 (10)

**Table 2:** Hydrogen-bond geometry (Å, °) *Cg*1 and *Cg*2 are the centroids of the C8*B*–C13*B* and C29*A*–C34*A* rings, respectively.

*D*—H⋯*A*	*D*—H	H⋯*A*	*D*⋯*A*	*D*—H⋯*A*
C5*A*—H5*AA*⋯O1*A* ^i^	0.95	2.55	3.221 (5)	127
C10*B*—H10*B*⋯O1*B* ^ii^	0.95	2.58	3.250 (5)	128
C3*B*—H3*BA*⋯*Cg*1	0.95	2.78	3.485 (4)	131
C12*B*—H12*B*⋯*Cg*2^iii^	0.95	2.67	3.539 (4)	153

Symmetry codes: (i) 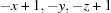; (ii) 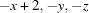; (iii) 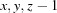.

**Fig. 1.**
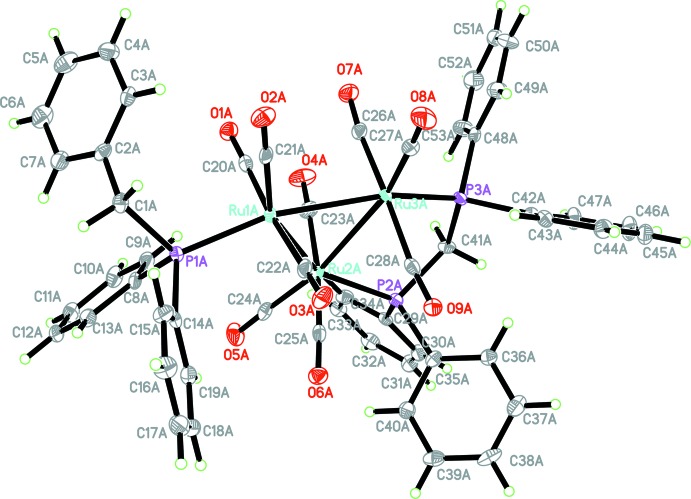
Molecule A the title compound, showing 50% probability displacement ellipsoids.

**Fig. 2.**
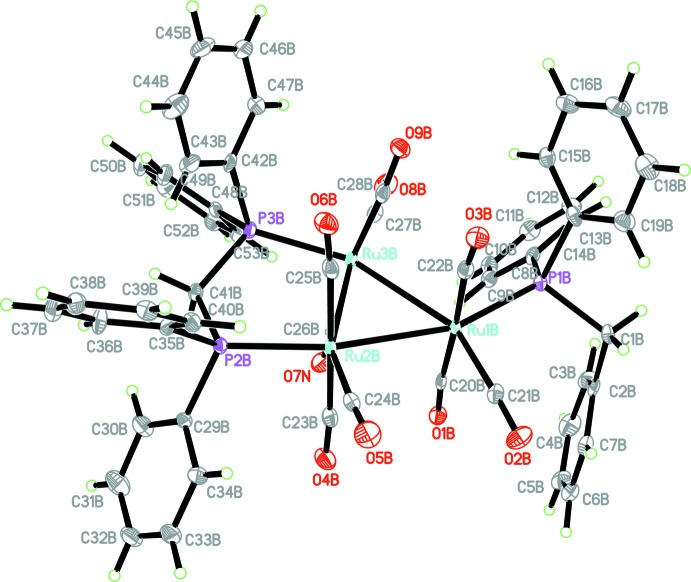
Molecule B the title compound, showing 50% probability displacement ellipsoids.

**Fig. 3.**
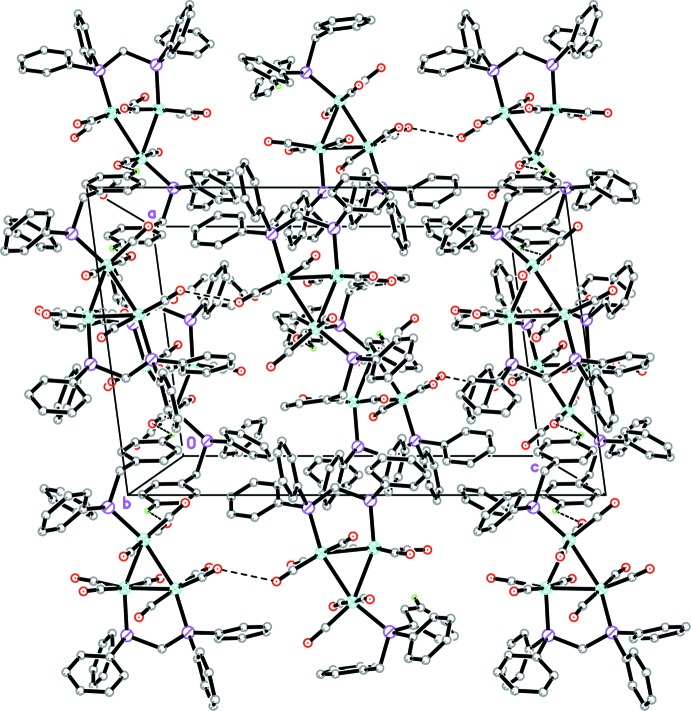
The crystal packing of (I). Dashed lines indicate hydrogen bonds. H atoms not involved in the hydrogen bond interactions have been omitted for clarity.

## supplementary crystallographic information

### S1. Comment

The synthesis and structural reports on substituted triruthenium clusters towards group 15 ligands are of interest as these ligands can often be used to stabilize metal clusters (Bruce *et al.*, 1988*a*,*b*). In the title compound, each Ru atom carries one equatorial and two axial terminal carbonyl ligands (Figures 1 & 2, Table 1). The bond lengths and angles are normal and correspond to those observed in related structures Koutsantonis *et al.* (2002), Shawkataly *et al.* (1998, 2009, 2010, 2011, 2012). The Ru–Ru bond *cis* to the P(C_19_H_17_) ligand is the longest Ru—Ru distance [Ru1A—Ru2A = 2.9054 (4) Å, Ru1B—Ru3B = 2.8992 (4) Å] in both molecules when compared to the other Ru—Ru distances [Ru1A—Ru3A = 2.8454 (4) Å and Ru2A—Ru3A = 2.8353 (4) Å]; [Ru1B—Ru2B = 2.8504 (4) and Ru2B—Ru3B = 2.8451 (4) Å]. The two phenyl rings (C8—C13 and C14—C19) of the benzyldiphenylphosphane ligand bound to the P1 atom make dihedral angles of 64.7 (2) and 68.6 (2)° with one another in the two molecules. They also subtend dihedral angles of 47.2 (2) & 59.8 (2) or 40.0 (2) & 46.3 (2)° respectively for molecules A and B with the (C2—C7) benzene rings of the benzyl groups. In the dppm ligand, the two benzene rings attached to each P2 (C29—C34 and C35—C40) and P3 (C42—C47 and C48—C53) phosphorus atoms make dihedral angles of 72.1 (2) and 80.2 (2)° with one another for molecule A and 86.9 (2) and 75.7 (2)° for molecule B.

In the crystal structure, molecules are linked by C5A—H5A···O1A and C10B—H10B···O1B hydrogen bonds, Fig.2, to form two dimensional layers parallel to the *ac* plane. C—H···π contacts, Table 2, further stabilise the crystal packing.

### S2. Experimental

All manipulations were performed under a dry oxygen-free nitrogen atmosphere using standard Schlenk technique. The Ru_3_(CO)_12_ (Aldrich), P(C_19_H_17_) (Strem Chemicals) and bis(diphenylphosphanyl)methane (Aldrich) were used as received. Ru_3_(Ph_2_PCH_2_PPh_2_)(CO)_10_ was prepared from Ru_3_(CO)_12_ and Ph_2_PCH_2_PPh_2_ using the benzophenone ketyl radical under mild conditions (Bruce *et al.* 1983). The title compound was then obtained by refluxing equimolar quantities of Ru_3_(Ph_2_PCH_2_PPh_2_)(CO)_10_ with P(C_19_H_17_) in n-hexane under a nitrogen atmosphere. Crystal suitable for X-ray diffraction were grown by solvent/solvent diffusion of CH_3_OH into CH_2_Cl_2_.

### S3. Refinement

All H atoms were positioned geometrically and refined using a riding model with with C–H = 0.95–0.99 Å and *U*_iso_(H) = 1.2*U*_eq_(C).

### Crystal data

**Table:**

[Ru_3_(C_19_H_17_P)(C_25_H_22_P_2_)(CO)_9_]	*F*(000) = 4848
*M**_r_* = 1215.96	*D*_x_ = 1.656 Mg m^−^^3^
Monoclinic, *P*2_1_/*n*	Mo *K*α radiation, λ = 0.71073 Å
Hall symbol: -P 2yn	Cell parameters from 9920 reflections
*a* = 13.4308 (2) Å	θ = 2.3–30.2°
*b* = 35.3969 (6) Å	µ = 1.07 mm^−^^1^
*c* = 20.6967 (3) Å	*T* = 100 K
β = 97.620 (1)°	Plate, red
*V* = 9752.5 (3) Å^3^	0.51 × 0.14 × 0.05 mm
*Z* = 8	

### Data collection

**Table:**

Bruker SMART APEXII CCD area-detector diffractometer	22388 independent reflections
Radiation source: fine-focus sealed tube	17595 reflections with *I* > 2σ(*I*)
Graphite monochromator	*R*_int_ = 0.058
φ and ω scans	θ_max_ = 27.5°, θ_min_ = 1.2°
Absorption correction: multi-scan (*SADABS*; Bruker, 2009)	*h* = −17→17
*T*_min_ = 0.612, *T*_max_ = 0.950	*k* = −45→45
96674 measured reflections	*l* = −26→26

### Refinement

**Table:**

Refinement on *F*^2^	Primary atom site location: structure-invariant direct methods
Least-squares matrix: full	Secondary atom site location: difference Fourier map
*R*[*F*^2^ > 2σ(*F*^2^)] = 0.043	Hydrogen site location: inferred from neighbouring sites
*wR*(*F*^2^) = 0.089	H-atom parameters constrained
*S* = 1.06	*w* = 1/[σ^2^(*F*_o_^2^) + (0.0272*P*)^2^ + 21.4411*P*] where *P* = (*F*_o_^2^ + 2*F*_c_^2^)/3
22388 reflections	(Δ/σ)_max_ = 0.002
1225 parameters	Δρ_max_ = 1.96 e Å^−^^3^
0 restraints	Δρ_min_ = −0.61 e Å^−^^3^

### Special details

**Table:**

Geometry. All e.s.d.'s (except the e.s.d. in the dihedral angle between two l.s. planes) are estimated using the full covariance matrix. The cell e.s.d.'s are taken into account individually in the estimation of e.s.d.'s in distances, angles and torsion angles; correlations between e.s.d.'s in cell parameters are only used when they are defined by crystal symmetry. An approximate (isotropic) treatment of cell e.s.d.'s is used for estimating e.s.d.'s involving l.s. planes.
Refinement. Refinement of *F*^2^ against ALL reflections. The weighted *R*-factor *wR* and goodness of fit *S* are based on *F*^2^, conventional *R*-factors *R* are based on *F*, with *F* set to zero for negative *F*^2^. The threshold expression of *F*^2^ > σ(*F*^2^) is used only for calculating *R*-factors(gt) *etc*. and is not relevant to the choice of reflections for refinement. *R*-factors based on *F*^2^ are statistically about twice as large as those based on *F*, and *R*- factors based on ALL data will be even larger.

### Fractional atomic coordinates and isotropic or equivalent isotropic displacement parameters (Å^2^)

**Table:**

	*x*	*y*	*z*	*U*_iso_*/*U*_eq_	
Ru1A	0.72460 (2)	0.138334 (8)	0.539334 (13)	0.01007 (6)	
Ru2A	0.90993 (2)	0.106019 (9)	0.604698 (13)	0.01080 (7)	
Ru3A	0.90419 (2)	0.140114 (9)	0.480298 (13)	0.01118 (7)	
P1A	0.60626 (7)	0.13535 (3)	0.61229 (4)	0.01132 (19)	
P2A	1.08419 (7)	0.10615 (3)	0.61365 (4)	0.01167 (19)	
P3A	1.06732 (7)	0.12496 (3)	0.46467 (4)	0.01180 (19)	
O1A	0.6812 (2)	0.05736 (8)	0.49014 (13)	0.0190 (6)	
O2A	0.5845 (2)	0.16919 (8)	0.42524 (13)	0.0232 (7)	
O3A	0.7657 (2)	0.22181 (8)	0.57580 (14)	0.0213 (6)	
O4A	0.8945 (2)	0.03101 (8)	0.52916 (14)	0.0283 (7)	
O5A	0.8844 (2)	0.06222 (9)	0.72792 (14)	0.0279 (7)	
O6A	0.9021 (2)	0.17726 (8)	0.68794 (13)	0.0188 (6)	
O7A	0.8433 (2)	0.06813 (8)	0.40158 (14)	0.0249 (7)	
O8A	0.8176 (2)	0.19088 (9)	0.36830 (14)	0.0294 (7)	
O9A	0.9857 (2)	0.20861 (8)	0.56152 (13)	0.0198 (6)	
C1A	0.4736 (3)	0.13267 (11)	0.57326 (19)	0.0165 (8)	
H1AA	0.4292	0.1372	0.6071	0.020*	
H1AB	0.4616	0.1533	0.5409	0.020*	
C2A	0.4433 (3)	0.09552 (11)	0.53939 (18)	0.0154 (8)	
C3A	0.4421 (3)	0.09125 (11)	0.47277 (19)	0.0167 (8)	
H3AA	0.4650	0.1113	0.4480	0.020*	
C4A	0.4077 (3)	0.05795 (12)	0.4416 (2)	0.0233 (9)	
H4AA	0.4053	0.0556	0.3957	0.028*	
C5A	0.3770 (4)	0.02826 (13)	0.4777 (2)	0.0284 (10)	
H5AA	0.3540	0.0055	0.4566	0.034*	
C6A	0.3798 (3)	0.03178 (12)	0.5446 (2)	0.0267 (10)	
H6AA	0.3589	0.0114	0.5694	0.032*	
C7A	0.4132 (3)	0.06505 (12)	0.5750 (2)	0.0206 (9)	
H7AA	0.4157	0.0672	0.6210	0.025*	
C8A	0.6067 (3)	0.09785 (11)	0.67336 (17)	0.0135 (8)	
C9A	0.6511 (3)	0.06318 (11)	0.66451 (19)	0.0168 (8)	
H9AA	0.6862	0.0594	0.6281	0.020*	
C10A	0.6446 (3)	0.03396 (12)	0.7085 (2)	0.0204 (9)	
H10A	0.6747	0.0102	0.7019	0.025*	
C11A	0.5948 (3)	0.03925 (12)	0.76183 (19)	0.0207 (9)	
H11A	0.5917	0.0194	0.7923	0.025*	
C12A	0.5492 (3)	0.07369 (12)	0.77108 (18)	0.0203 (9)	
H12A	0.5144	0.0773	0.8077	0.024*	
C13A	0.5543 (3)	0.10282 (12)	0.72690 (18)	0.0175 (8)	
H13A	0.5222	0.1262	0.7330	0.021*	
C14A	0.6054 (3)	0.17780 (11)	0.66222 (18)	0.0148 (8)	
C15A	0.5429 (3)	0.20827 (11)	0.64259 (19)	0.0185 (8)	
H15A	0.4971	0.2065	0.6036	0.022*	
C16A	0.5467 (3)	0.24122 (12)	0.6795 (2)	0.0226 (9)	
H16A	0.5030	0.2616	0.6660	0.027*	
C17A	0.6145 (3)	0.24419 (12)	0.7359 (2)	0.0232 (9)	
H17A	0.6174	0.2667	0.7610	0.028*	
C18A	0.6780 (3)	0.21432 (12)	0.75574 (19)	0.0201 (9)	
H18A	0.7247	0.2164	0.7943	0.024*	
C19A	0.6731 (3)	0.18144 (12)	0.71899 (18)	0.0172 (8)	
H19A	0.7167	0.1610	0.7328	0.021*	
C20A	0.7031 (3)	0.08688 (11)	0.51039 (17)	0.0140 (8)	
C21A	0.6377 (3)	0.15730 (11)	0.46847 (18)	0.0143 (8)	
C22A	0.7568 (3)	0.19032 (11)	0.56464 (18)	0.0155 (8)	
C23A	0.8975 (3)	0.06004 (12)	0.55399 (19)	0.0186 (9)	
C24A	0.8915 (3)	0.07910 (11)	0.68156 (19)	0.0169 (8)	
C25A	0.9020 (3)	0.15224 (11)	0.65249 (18)	0.0153 (8)	
C26A	0.8625 (3)	0.09425 (12)	0.43299 (18)	0.0167 (8)	
C27A	0.8528 (3)	0.17142 (12)	0.40919 (18)	0.0185 (9)	
C28A	0.9525 (3)	0.18268 (11)	0.53423 (17)	0.0154 (8)	
C29A	1.1497 (3)	0.06687 (11)	0.65981 (17)	0.0131 (8)	
C30A	1.2479 (3)	0.07002 (12)	0.69070 (18)	0.0163 (8)	
H30A	1.2827	0.0933	0.6892	0.020*	
C31A	1.2954 (3)	0.03952 (12)	0.72368 (19)	0.0200 (9)	
H31A	1.3621	0.0422	0.7451	0.024*	
C32A	1.2467 (3)	0.00529 (12)	0.72574 (19)	0.0204 (9)	
H32A	1.2798	−0.0156	0.7480	0.024*	
C33A	1.1490 (3)	0.00165 (11)	0.69510 (18)	0.0180 (8)	
H33A	1.1149	−0.0218	0.6964	0.022*	
C34A	1.1006 (3)	0.03221 (11)	0.66248 (18)	0.0173 (8)	
H34A	1.0335	0.0295	0.6418	0.021*	
C35A	1.1490 (3)	0.14688 (11)	0.65245 (18)	0.0136 (8)	
C36A	1.2073 (3)	0.17161 (12)	0.62093 (19)	0.0187 (9)	
H36A	1.2140	0.1677	0.5763	0.022*	
C37A	1.2559 (3)	0.20199 (12)	0.6542 (2)	0.0240 (9)	
H37A	1.2944	0.2189	0.6319	0.029*	
C38A	1.2487 (3)	0.20764 (12)	0.7193 (2)	0.0241 (10)	
H38A	1.2827	0.2282	0.7419	0.029*	
C39A	1.1913 (3)	0.18314 (12)	0.75164 (19)	0.0205 (9)	
H39A	1.1869	0.1867	0.7967	0.025*	
C40A	1.1408 (3)	0.15377 (11)	0.71835 (18)	0.0174 (8)	
H40A	1.0996	0.1378	0.7404	0.021*	
C41A	1.1356 (3)	0.09986 (11)	0.53572 (16)	0.0139 (8)	
H41A	1.1360	0.0725	0.5256	0.017*	
H41B	1.2063	0.1086	0.5417	0.017*	
C42A	1.1501 (3)	0.16317 (11)	0.44539 (17)	0.0147 (8)	
C43A	1.1139 (3)	0.19955 (11)	0.43306 (18)	0.0168 (8)	
H43A	1.0457	0.2051	0.4368	0.020*	
C44A	1.1767 (3)	0.22808 (12)	0.41526 (19)	0.0192 (9)	
H44A	1.1511	0.2529	0.4067	0.023*	
C45A	1.2761 (3)	0.22019 (12)	0.41019 (19)	0.0210 (9)	
H45A	1.3194	0.2397	0.3992	0.025*	
C46A	1.3126 (3)	0.18380 (13)	0.4211 (2)	0.0252 (10)	
H46A	1.3806	0.1783	0.4168	0.030*	
C47A	1.2500 (3)	0.15534 (12)	0.43837 (19)	0.0204 (9)	
H47A	1.2753	0.1304	0.4454	0.024*	
C48A	1.0805 (3)	0.09275 (11)	0.39672 (18)	0.0150 (8)	
C49A	1.0541 (3)	0.10695 (13)	0.33411 (19)	0.0235 (9)	
H49A	1.0323	0.1324	0.3286	0.028*	
C50A	1.0591 (4)	0.08461 (14)	0.2798 (2)	0.0287 (11)	
H50A	1.0412	0.0948	0.2374	0.034*	
C51A	1.0902 (3)	0.04710 (14)	0.2872 (2)	0.0276 (10)	
H51A	1.0922	0.0315	0.2501	0.033*	
C52A	1.1182 (4)	0.03287 (13)	0.3491 (2)	0.0266 (10)	
H52A	1.1413	0.0076	0.3544	0.032*	
C53A	1.1128 (3)	0.05540 (12)	0.4039 (2)	0.0218 (9)	
H53A	1.1312	0.0452	0.4463	0.026*	
Ru1B	0.95027 (2)	0.141361 (8)	0.065726 (13)	0.00935 (6)	
Ru2B	0.77097 (2)	0.149707 (9)	0.124216 (13)	0.01061 (7)	
Ru3B	0.75930 (2)	0.114774 (8)	−0.000365 (13)	0.01013 (6)	
P1B	1.06230 (7)	0.13458 (3)	−0.01102 (4)	0.01099 (19)	
P2B	0.60760 (7)	0.13624 (3)	0.14157 (4)	0.01093 (19)	
P3B	0.58565 (7)	0.11496 (3)	−0.00632 (4)	0.01164 (19)	
O1B	0.9909 (2)	0.06047 (8)	0.11646 (13)	0.0166 (6)	
O2B	1.0914 (2)	0.16740 (8)	0.18338 (13)	0.0228 (6)	
O3B	0.9172 (2)	0.22463 (8)	0.02485 (13)	0.0197 (6)	
O4B	0.8322 (2)	0.07807 (9)	0.20321 (14)	0.0254 (7)	
O5B	0.8601 (2)	0.20092 (9)	0.23486 (14)	0.0299 (8)	
O6B	0.6945 (2)	0.21945 (8)	0.04407 (13)	0.0218 (6)	
O7N	0.7780 (2)	0.03940 (8)	0.07390 (14)	0.0248 (7)	
O8B	0.7763 (2)	0.07413 (9)	−0.12786 (14)	0.0269 (7)	
O9B	0.7655 (2)	0.18688 (8)	−0.08184 (13)	0.0187 (6)	
C1B	1.1966 (3)	0.12882 (11)	0.02456 (18)	0.0165 (8)	
H1BA	1.2388	0.1287	−0.0112	0.020*	
H1BB	1.2170	0.1508	0.0528	0.020*	
C2B	1.2164 (3)	0.09327 (11)	0.06379 (18)	0.0153 (8)	
C3B	1.2189 (3)	0.05801 (12)	0.0341 (2)	0.0197 (9)	
H3BA	1.2144	0.0564	−0.0120	0.024*	
C4B	1.2277 (3)	0.02531 (12)	0.0709 (2)	0.0251 (10)	
H4BA	1.2254	0.0015	0.0497	0.030*	
C5B	1.2400 (3)	0.02696 (13)	0.1386 (2)	0.0249 (10)	
H5BA	1.2456	0.0044	0.1637	0.030*	
C6B	1.2438 (3)	0.06168 (13)	0.1687 (2)	0.0239 (10)	
H6BA	1.2556	0.0632	0.2149	0.029*	
C7B	1.2304 (3)	0.09446 (12)	0.13185 (19)	0.0182 (8)	
H7BA	1.2309	0.1182	0.1533	0.022*	
C8B	1.0501 (3)	0.09557 (11)	−0.06920 (18)	0.0133 (8)	
C9B	1.0016 (3)	0.06219 (11)	−0.05504 (18)	0.0147 (8)	
H9BA	0.9663	0.0613	−0.0182	0.018*	
C10B	1.0042 (3)	0.03033 (12)	−0.09388 (19)	0.0183 (8)	
H10B	0.9724	0.0077	−0.0830	0.022*	
C11B	1.0535 (3)	0.03176 (12)	−0.14880 (19)	0.0204 (9)	
H11B	1.0554	0.0100	−0.1755	0.024*	
C12B	1.1000 (3)	0.06507 (12)	−0.16466 (18)	0.0188 (9)	
H12B	1.1320	0.0663	−0.2029	0.023*	
C13B	1.0996 (3)	0.09638 (12)	−0.12477 (17)	0.0160 (8)	
H13B	1.1333	0.1187	−0.1351	0.019*	
C14B	1.0666 (3)	0.17601 (11)	−0.06273 (18)	0.0144 (8)	
C15B	0.9946 (3)	0.18015 (11)	−0.11765 (18)	0.0161 (8)	
H15B	0.9486	0.1602	−0.1298	0.019*	
C16B	0.9894 (3)	0.21294 (12)	−0.15472 (19)	0.0199 (9)	
H16B	0.9400	0.2153	−0.1919	0.024*	
C17B	1.0561 (3)	0.24223 (12)	−0.1375 (2)	0.0229 (9)	
H17B	1.0523	0.2648	−0.1626	0.028*	
C18B	1.1283 (3)	0.23856 (12)	−0.0836 (2)	0.0240 (9)	
H18B	1.1746	0.2585	−0.0720	0.029*	
C19B	1.1334 (3)	0.20558 (12)	−0.0461 (2)	0.0210 (9)	
H19B	1.1828	0.2033	−0.0090	0.025*	
C20B	0.9702 (3)	0.08984 (11)	0.09573 (17)	0.0121 (8)	
C21B	1.0387 (3)	0.15807 (10)	0.13762 (18)	0.0139 (8)	
C22B	0.9234 (3)	0.19355 (11)	0.03834 (18)	0.0147 (8)	
C23B	0.8131 (3)	0.10392 (12)	0.17163 (19)	0.0189 (9)	
C24B	0.8253 (3)	0.18124 (12)	0.19488 (18)	0.0173 (8)	
C25B	0.7259 (3)	0.19271 (12)	0.07068 (18)	0.0157 (8)	
C26B	0.7733 (3)	0.06860 (12)	0.04949 (18)	0.0173 (8)	
C27B	0.7730 (3)	0.08942 (11)	−0.07923 (19)	0.0167 (8)	
C28B	0.7662 (3)	0.16171 (11)	−0.04736 (17)	0.0145 (8)	
C29B	0.5948 (3)	0.10651 (11)	0.21202 (17)	0.0134 (8)	
C30B	0.5342 (3)	0.07484 (12)	0.21128 (19)	0.0217 (9)	
H30B	0.4964	0.0669	0.1715	0.026*	
C31B	0.5280 (4)	0.05449 (13)	0.2683 (2)	0.0283 (10)	
H31B	0.4875	0.0324	0.2670	0.034*	
C32B	0.5804 (3)	0.06624 (13)	0.3264 (2)	0.0259 (10)	
H32B	0.5766	0.0521	0.3651	0.031*	
C33B	0.6382 (3)	0.09836 (13)	0.32857 (19)	0.0233 (9)	
H33B	0.6730	0.1067	0.3690	0.028*	
C34B	0.6458 (3)	0.11862 (12)	0.27211 (17)	0.0183 (8)	
H34B	0.6858	0.1408	0.2741	0.022*	
C35B	0.5234 (3)	0.17437 (11)	0.15831 (17)	0.0124 (7)	
C36B	0.4232 (3)	0.16650 (12)	0.1637 (2)	0.0189 (8)	
H36B	0.3984	0.1415	0.1563	0.023*	
C37B	0.3592 (3)	0.19484 (12)	0.17970 (19)	0.0207 (9)	
H37B	0.2909	0.1892	0.1829	0.025*	
C38B	0.3950 (3)	0.23121 (12)	0.19101 (18)	0.0175 (8)	
H38B	0.3510	0.2507	0.2014	0.021*	
C39B	0.4948 (3)	0.23925 (11)	0.18723 (18)	0.0170 (8)	
H39B	0.5197	0.2641	0.1960	0.020*	
C40B	0.5586 (3)	0.21108 (11)	0.17060 (17)	0.0150 (8)	
H40B	0.6269	0.2169	0.1676	0.018*	
C41B	0.5392 (3)	0.10925 (11)	0.07339 (17)	0.0121 (7)	
H41C	0.4677	0.1169	0.0684	0.015*	
H41D	0.5423	0.0821	0.0851	0.015*	
C42B	0.5198 (3)	0.15548 (11)	−0.04562 (18)	0.0145 (8)	
C43B	0.4648 (3)	0.18089 (12)	−0.01374 (19)	0.0192 (9)	
H43B	0.4598	0.1774	0.0312	0.023*	
C44B	0.4166 (3)	0.21164 (12)	−0.0470 (2)	0.0251 (10)	
H44B	0.3801	0.2291	−0.0245	0.030*	
C45B	0.4223 (3)	0.21653 (12)	−0.1132 (2)	0.0240 (9)	
H45B	0.3887	0.2371	−0.1362	0.029*	
C46B	0.4773 (3)	0.19118 (12)	−0.1454 (2)	0.0228 (9)	
H46B	0.4812	0.1944	−0.1905	0.027*	
C47B	0.5265 (3)	0.16125 (12)	−0.11224 (18)	0.0181 (8)	
H47B	0.5651	0.1444	−0.1346	0.022*	
C48B	0.5174 (3)	0.07522 (11)	−0.04912 (17)	0.0135 (8)	
C49B	0.4157 (3)	0.07783 (12)	−0.07362 (19)	0.0198 (9)	
H49B	0.3809	0.1010	−0.0702	0.024*	
C50B	0.3648 (3)	0.04685 (13)	−0.1030 (2)	0.0240 (10)	
H50B	0.2958	0.0490	−0.1200	0.029*	
C51B	0.4146 (3)	0.01286 (12)	−0.10756 (18)	0.0217 (9)	
H51B	0.3797	−0.0083	−0.1278	0.026*	
C52B	0.5151 (3)	0.00967 (12)	−0.08257 (19)	0.0198 (9)	
H52B	0.5493	−0.0136	−0.0853	0.024*	
C53B	0.5654 (3)	0.04081 (11)	−0.05346 (18)	0.0164 (8)	
H53B	0.6343	0.0385	−0.0361	0.020*	

### Atomic displacement parameters (Å^2^)

**Table:**

	*U*^11^	*U*^22^	*U*^33^	*U*^12^	*U*^13^	*U*^23^
Ru1A	0.00897 (15)	0.01017 (14)	0.01122 (13)	0.00034 (12)	0.00190 (11)	0.00117 (11)
Ru2A	0.00956 (15)	0.01208 (15)	0.01064 (13)	0.00028 (12)	0.00093 (11)	0.00143 (11)
Ru3A	0.00985 (15)	0.01421 (15)	0.00964 (13)	0.00038 (12)	0.00186 (11)	0.00057 (11)
P1A	0.0110 (5)	0.0106 (5)	0.0127 (4)	0.0003 (4)	0.0029 (4)	0.0005 (4)
P2A	0.0109 (5)	0.0128 (5)	0.0112 (4)	0.0015 (4)	0.0011 (4)	0.0002 (4)
P3A	0.0104 (5)	0.0156 (5)	0.0096 (4)	0.0007 (4)	0.0022 (4)	−0.0001 (4)
O1A	0.0198 (16)	0.0153 (15)	0.0220 (14)	−0.0020 (12)	0.0030 (12)	−0.0018 (12)
O2A	0.0228 (16)	0.0214 (16)	0.0227 (15)	0.0000 (13)	−0.0069 (13)	0.0055 (12)
O3A	0.0196 (16)	0.0150 (16)	0.0291 (16)	−0.0028 (12)	0.0020 (12)	−0.0020 (12)
O4A	0.038 (2)	0.0155 (16)	0.0276 (16)	0.0026 (14)	−0.0094 (14)	−0.0023 (13)
O5A	0.0244 (17)	0.0380 (19)	0.0213 (15)	−0.0017 (15)	0.0032 (13)	0.0149 (14)
O6A	0.0179 (15)	0.0184 (15)	0.0199 (14)	0.0009 (12)	0.0020 (11)	−0.0039 (12)
O7A	0.0243 (17)	0.0259 (17)	0.0249 (15)	−0.0053 (14)	0.0051 (13)	−0.0101 (13)
O8A	0.0332 (19)	0.0349 (19)	0.0187 (15)	0.0028 (15)	−0.0019 (13)	0.0114 (13)
O9A	0.0217 (16)	0.0200 (15)	0.0182 (14)	−0.0043 (13)	0.0051 (12)	−0.0042 (12)
C1A	0.014 (2)	0.016 (2)	0.0205 (19)	0.0014 (16)	0.0052 (15)	0.0004 (15)
C2A	0.0095 (19)	0.015 (2)	0.0205 (19)	0.0022 (16)	−0.0017 (15)	−0.0025 (15)
C3A	0.013 (2)	0.016 (2)	0.0214 (19)	−0.0024 (16)	0.0018 (15)	0.0036 (16)
C4A	0.024 (2)	0.027 (2)	0.019 (2)	0.0002 (19)	0.0020 (17)	−0.0041 (17)
C5A	0.030 (3)	0.018 (2)	0.035 (2)	−0.008 (2)	−0.002 (2)	−0.0049 (19)
C6A	0.028 (3)	0.020 (2)	0.031 (2)	−0.006 (2)	0.0000 (19)	0.0075 (18)
C7A	0.018 (2)	0.022 (2)	0.021 (2)	−0.0025 (18)	0.0010 (16)	0.0032 (17)
C8A	0.0101 (19)	0.016 (2)	0.0148 (17)	−0.0020 (15)	0.0023 (14)	0.0031 (15)
C9A	0.013 (2)	0.020 (2)	0.0181 (19)	−0.0003 (16)	0.0014 (15)	0.0014 (16)
C10A	0.017 (2)	0.016 (2)	0.028 (2)	−0.0006 (17)	0.0022 (17)	0.0075 (17)
C11A	0.019 (2)	0.024 (2)	0.0180 (19)	−0.0058 (18)	−0.0014 (16)	0.0110 (17)
C12A	0.019 (2)	0.029 (2)	0.0134 (18)	−0.0091 (18)	0.0045 (16)	0.0018 (16)
C13A	0.016 (2)	0.017 (2)	0.0198 (19)	−0.0015 (17)	0.0025 (16)	−0.0007 (16)
C14A	0.014 (2)	0.017 (2)	0.0146 (18)	−0.0007 (16)	0.0071 (15)	0.0016 (15)
C15A	0.016 (2)	0.019 (2)	0.0190 (19)	−0.0011 (17)	−0.0011 (16)	−0.0022 (16)
C16A	0.026 (2)	0.015 (2)	0.027 (2)	0.0027 (18)	0.0039 (18)	−0.0008 (17)
C17A	0.031 (3)	0.018 (2)	0.022 (2)	−0.0072 (19)	0.0099 (18)	−0.0029 (17)
C18A	0.023 (2)	0.022 (2)	0.0161 (19)	−0.0070 (18)	0.0033 (16)	−0.0006 (16)
C19A	0.015 (2)	0.019 (2)	0.0177 (18)	−0.0018 (17)	0.0042 (16)	0.0008 (16)
C20A	0.0106 (19)	0.018 (2)	0.0134 (17)	0.0028 (16)	0.0025 (14)	0.0055 (15)
C21A	0.014 (2)	0.0130 (19)	0.0168 (18)	−0.0048 (16)	0.0033 (15)	0.0021 (15)
C22A	0.0089 (19)	0.020 (2)	0.0185 (19)	−0.0004 (16)	0.0030 (15)	0.0009 (16)
C23A	0.016 (2)	0.019 (2)	0.0192 (19)	0.0020 (17)	−0.0052 (16)	0.0045 (16)
C24A	0.0097 (19)	0.017 (2)	0.023 (2)	−0.0013 (16)	0.0003 (15)	−0.0001 (16)
C25A	0.0106 (19)	0.019 (2)	0.0165 (18)	−0.0004 (16)	0.0037 (15)	0.0039 (16)
C26A	0.012 (2)	0.021 (2)	0.0161 (18)	−0.0015 (17)	0.0002 (15)	0.0035 (16)
C27A	0.015 (2)	0.024 (2)	0.0165 (19)	−0.0024 (17)	0.0014 (16)	−0.0023 (16)
C28A	0.014 (2)	0.020 (2)	0.0128 (17)	0.0027 (17)	0.0052 (15)	0.0014 (15)
C29A	0.0124 (19)	0.018 (2)	0.0094 (16)	0.0042 (16)	0.0029 (14)	−0.0008 (14)
C30A	0.011 (2)	0.023 (2)	0.0147 (18)	0.0014 (17)	0.0003 (15)	0.0046 (16)
C31A	0.011 (2)	0.028 (2)	0.0195 (19)	0.0039 (17)	−0.0011 (16)	0.0028 (17)
C32A	0.020 (2)	0.023 (2)	0.0173 (19)	0.0104 (18)	0.0020 (16)	0.0024 (16)
C33A	0.024 (2)	0.012 (2)	0.0179 (18)	0.0007 (17)	0.0040 (17)	0.0003 (15)
C34A	0.017 (2)	0.020 (2)	0.0145 (18)	0.0029 (17)	−0.0021 (15)	0.0000 (15)
C35A	0.0078 (18)	0.014 (2)	0.0176 (18)	0.0028 (15)	−0.0023 (14)	0.0010 (15)
C36A	0.018 (2)	0.022 (2)	0.0154 (18)	−0.0035 (17)	0.0001 (16)	0.0003 (16)
C37A	0.021 (2)	0.022 (2)	0.028 (2)	−0.0048 (19)	−0.0021 (18)	0.0035 (18)
C38A	0.021 (2)	0.018 (2)	0.030 (2)	−0.0005 (18)	−0.0088 (18)	−0.0076 (18)
C39A	0.020 (2)	0.022 (2)	0.0180 (19)	0.0032 (18)	−0.0036 (16)	−0.0034 (16)
C40A	0.014 (2)	0.020 (2)	0.0184 (19)	0.0012 (17)	0.0014 (15)	−0.0003 (16)
C41A	0.0129 (19)	0.017 (2)	0.0110 (17)	0.0055 (16)	−0.0018 (14)	−0.0007 (14)
C42A	0.014 (2)	0.018 (2)	0.0115 (17)	−0.0034 (16)	0.0016 (14)	−0.0012 (15)
C43A	0.018 (2)	0.019 (2)	0.0146 (18)	−0.0014 (17)	0.0055 (15)	−0.0015 (15)
C44A	0.021 (2)	0.017 (2)	0.0195 (19)	−0.0007 (17)	0.0017 (16)	0.0013 (16)
C45A	0.019 (2)	0.023 (2)	0.021 (2)	−0.0054 (18)	0.0031 (17)	0.0042 (17)
C46A	0.013 (2)	0.032 (3)	0.032 (2)	−0.0008 (19)	0.0058 (18)	0.0072 (19)
C47A	0.013 (2)	0.023 (2)	0.025 (2)	0.0018 (17)	0.0042 (16)	0.0086 (17)
C48A	0.0092 (19)	0.021 (2)	0.0152 (18)	−0.0027 (16)	0.0044 (14)	−0.0032 (15)
C49A	0.027 (2)	0.024 (2)	0.0185 (19)	−0.0018 (19)	0.0008 (17)	−0.0018 (17)
C50A	0.034 (3)	0.040 (3)	0.0118 (19)	−0.006 (2)	0.0017 (18)	−0.0033 (18)
C51A	0.025 (2)	0.037 (3)	0.023 (2)	−0.014 (2)	0.0118 (18)	−0.0149 (19)
C52A	0.032 (3)	0.021 (2)	0.029 (2)	−0.002 (2)	0.010 (2)	−0.0087 (18)
C53A	0.026 (2)	0.020 (2)	0.019 (2)	0.0025 (18)	0.0031 (17)	−0.0038 (16)
Ru1B	0.00733 (14)	0.00995 (14)	0.01097 (13)	−0.00050 (12)	0.00192 (11)	−0.00086 (11)
Ru2B	0.00823 (15)	0.01379 (15)	0.01019 (13)	−0.00051 (12)	0.00259 (11)	−0.00082 (11)
Ru3B	0.00708 (14)	0.01262 (15)	0.01073 (13)	−0.00025 (12)	0.00130 (11)	−0.00110 (11)
P1B	0.0095 (5)	0.0114 (5)	0.0126 (4)	−0.0003 (4)	0.0033 (4)	−0.0003 (4)
P2B	0.0091 (5)	0.0128 (5)	0.0111 (4)	−0.0002 (4)	0.0023 (3)	−0.0013 (4)
P3B	0.0083 (5)	0.0148 (5)	0.0120 (4)	−0.0001 (4)	0.0019 (4)	−0.0011 (4)
O1B	0.0151 (15)	0.0147 (15)	0.0200 (13)	0.0003 (12)	0.0019 (11)	0.0014 (11)
O2B	0.0235 (17)	0.0186 (15)	0.0239 (15)	0.0002 (13)	−0.0057 (13)	−0.0056 (12)
O3B	0.0211 (16)	0.0133 (15)	0.0245 (15)	0.0024 (12)	0.0022 (12)	0.0033 (11)
O4B	0.0240 (17)	0.0272 (17)	0.0271 (16)	0.0105 (14)	0.0111 (13)	0.0122 (13)
O5B	0.0311 (19)	0.0370 (19)	0.0211 (15)	−0.0134 (15)	0.0013 (14)	−0.0114 (14)
O6B	0.0259 (17)	0.0207 (16)	0.0203 (14)	0.0053 (13)	0.0083 (12)	0.0021 (12)
O7N	0.0214 (17)	0.0198 (16)	0.0310 (16)	−0.0047 (13)	−0.0042 (13)	0.0085 (13)
O8B	0.0246 (17)	0.0347 (18)	0.0207 (15)	0.0043 (14)	0.0003 (13)	−0.0118 (13)
O9B	0.0195 (16)	0.0186 (15)	0.0186 (14)	0.0002 (12)	0.0051 (12)	0.0037 (12)
C1B	0.0097 (19)	0.022 (2)	0.0188 (19)	−0.0005 (16)	0.0058 (15)	0.0009 (16)
C2B	0.0046 (18)	0.021 (2)	0.0197 (19)	0.0015 (16)	0.0004 (14)	0.0037 (16)
C3B	0.013 (2)	0.024 (2)	0.021 (2)	0.0057 (17)	0.0009 (16)	0.0009 (17)
C4B	0.022 (2)	0.017 (2)	0.035 (2)	0.0064 (18)	0.0004 (19)	−0.0015 (18)
C5B	0.016 (2)	0.024 (2)	0.033 (2)	0.0043 (19)	−0.0023 (18)	0.0127 (19)
C6B	0.013 (2)	0.034 (3)	0.023 (2)	0.0000 (19)	−0.0009 (17)	0.0076 (18)
C7B	0.011 (2)	0.020 (2)	0.024 (2)	−0.0015 (16)	0.0017 (16)	−0.0014 (16)
C8B	0.0090 (18)	0.0139 (19)	0.0168 (18)	0.0026 (15)	0.0008 (14)	−0.0032 (15)
C9B	0.013 (2)	0.016 (2)	0.0156 (18)	0.0018 (16)	0.0027 (15)	−0.0030 (15)
C10B	0.016 (2)	0.017 (2)	0.0214 (19)	−0.0011 (17)	0.0030 (16)	−0.0024 (16)
C11B	0.023 (2)	0.019 (2)	0.0188 (19)	0.0035 (18)	0.0017 (17)	−0.0085 (16)
C12B	0.020 (2)	0.021 (2)	0.0158 (18)	0.0078 (18)	0.0043 (16)	0.0002 (16)
C13B	0.014 (2)	0.021 (2)	0.0136 (17)	0.0027 (16)	0.0025 (15)	0.0014 (15)
C14B	0.015 (2)	0.0133 (19)	0.0171 (18)	0.0009 (16)	0.0095 (15)	−0.0014 (15)
C15B	0.015 (2)	0.017 (2)	0.0178 (18)	0.0011 (16)	0.0057 (15)	−0.0012 (15)
C16B	0.025 (2)	0.019 (2)	0.0161 (19)	0.0040 (18)	0.0054 (17)	0.0016 (16)
C17B	0.033 (3)	0.014 (2)	0.023 (2)	0.0043 (19)	0.0083 (18)	0.0064 (16)
C18B	0.031 (3)	0.012 (2)	0.031 (2)	−0.0040 (18)	0.0067 (19)	−0.0003 (17)
C19B	0.022 (2)	0.019 (2)	0.022 (2)	0.0002 (18)	0.0037 (17)	0.0029 (16)
C20B	0.0060 (18)	0.018 (2)	0.0129 (17)	−0.0021 (15)	0.0031 (14)	−0.0045 (15)
C21B	0.0114 (19)	0.0097 (19)	0.0207 (19)	0.0018 (15)	0.0024 (15)	0.0018 (15)
C22B	0.0109 (19)	0.019 (2)	0.0144 (17)	0.0001 (16)	0.0020 (14)	−0.0029 (15)
C23B	0.014 (2)	0.026 (2)	0.0183 (19)	0.0020 (18)	0.0072 (16)	−0.0007 (17)
C24B	0.015 (2)	0.023 (2)	0.0152 (18)	−0.0013 (17)	0.0059 (15)	0.0025 (16)
C25B	0.014 (2)	0.020 (2)	0.0133 (17)	−0.0025 (17)	0.0058 (15)	−0.0050 (16)
C26B	0.0081 (19)	0.025 (2)	0.0166 (18)	−0.0008 (17)	−0.0045 (15)	−0.0023 (16)
C27B	0.012 (2)	0.018 (2)	0.0202 (19)	0.0009 (16)	0.0006 (15)	−0.0009 (16)
C28B	0.0090 (19)	0.021 (2)	0.0136 (17)	0.0016 (16)	0.0030 (14)	−0.0054 (16)
C29B	0.0126 (19)	0.018 (2)	0.0098 (16)	0.0030 (16)	0.0028 (14)	0.0000 (14)
C30B	0.025 (2)	0.023 (2)	0.0183 (19)	−0.0064 (19)	0.0064 (17)	−0.0014 (17)
C31B	0.037 (3)	0.022 (2)	0.029 (2)	−0.008 (2)	0.012 (2)	0.0029 (18)
C32B	0.027 (3)	0.033 (3)	0.020 (2)	0.007 (2)	0.0095 (18)	0.0100 (18)
C33B	0.023 (2)	0.034 (3)	0.0148 (19)	0.007 (2)	0.0065 (17)	−0.0001 (17)
C34B	0.019 (2)	0.024 (2)	0.0122 (17)	−0.0008 (18)	0.0021 (15)	−0.0021 (16)
C35B	0.0109 (19)	0.0154 (19)	0.0110 (16)	0.0016 (15)	0.0015 (14)	−0.0013 (14)
C36B	0.010 (2)	0.017 (2)	0.030 (2)	−0.0030 (16)	0.0041 (16)	−0.0029 (17)
C37B	0.014 (2)	0.025 (2)	0.023 (2)	0.0010 (18)	0.0033 (16)	−0.0052 (17)
C38B	0.017 (2)	0.022 (2)	0.0148 (18)	0.0059 (17)	0.0040 (15)	−0.0011 (15)
C39B	0.021 (2)	0.016 (2)	0.0142 (18)	0.0003 (17)	0.0033 (16)	0.0002 (15)
C40B	0.014 (2)	0.019 (2)	0.0126 (17)	−0.0008 (16)	0.0035 (15)	−0.0007 (15)
C41B	0.0099 (18)	0.0121 (19)	0.0144 (17)	−0.0014 (15)	0.0019 (14)	−0.0029 (14)
C42B	0.0064 (18)	0.016 (2)	0.0203 (19)	−0.0024 (15)	0.0000 (15)	0.0012 (15)
C43B	0.017 (2)	0.022 (2)	0.0179 (19)	0.0037 (17)	−0.0015 (16)	0.0005 (16)
C44B	0.024 (2)	0.020 (2)	0.030 (2)	0.0069 (19)	−0.0017 (19)	−0.0061 (18)
C45B	0.019 (2)	0.022 (2)	0.028 (2)	0.0027 (18)	−0.0069 (18)	0.0061 (18)
C46B	0.015 (2)	0.030 (2)	0.022 (2)	−0.0023 (18)	−0.0009 (17)	0.0073 (18)
C47B	0.016 (2)	0.022 (2)	0.0158 (18)	−0.0009 (17)	0.0030 (15)	0.0023 (16)
C48B	0.0107 (19)	0.019 (2)	0.0112 (17)	−0.0039 (16)	0.0023 (14)	0.0002 (14)
C49B	0.012 (2)	0.028 (2)	0.0201 (19)	0.0000 (17)	0.0018 (16)	−0.0043 (17)
C50B	0.011 (2)	0.038 (3)	0.023 (2)	−0.0025 (19)	0.0021 (17)	−0.0066 (19)
C51B	0.024 (2)	0.026 (2)	0.0152 (19)	−0.0090 (19)	0.0030 (17)	−0.0040 (16)
C52B	0.021 (2)	0.020 (2)	0.0184 (19)	−0.0043 (18)	0.0017 (16)	−0.0007 (16)
C53B	0.012 (2)	0.019 (2)	0.0169 (18)	−0.0037 (16)	−0.0018 (15)	−0.0010 (15)

### Geometric parameters (Å, º)

**Table:**

Ru1A—C21A	1.873 (4)	Ru1B—C21B	1.873 (4)
Ru1A—C20A	1.927 (4)	Ru1B—C20B	1.934 (4)
Ru1A—C22A	1.946 (4)	Ru1B—C22B	1.952 (4)
Ru1A—P1A	2.3353 (10)	Ru1B—P1B	2.3406 (10)
Ru1A—Ru3A	2.8454 (4)	Ru1B—Ru2B	2.8504 (4)
Ru1A—Ru2A	2.9054 (4)	Ru1B—Ru3B	2.8992 (4)
Ru2A—C24A	1.898 (4)	Ru2B—C24B	1.906 (4)
Ru2A—C25A	1.922 (4)	Ru2B—C25B	1.932 (4)
Ru2A—C23A	1.932 (4)	Ru2B—C23B	1.940 (4)
Ru2A—P2A	2.3232 (10)	Ru2B—P2B	2.3195 (10)
Ru2A—Ru3A	2.8353 (4)	Ru2B—Ru3B	2.8451 (4)
Ru3A—C27A	1.898 (4)	Ru3B—C27B	1.893 (4)
Ru3A—C28A	1.935 (4)	Ru3B—C26B	1.929 (4)
Ru3A—C26A	1.940 (4)	Ru3B—C28B	1.933 (4)
Ru3A—P3A	2.3196 (10)	Ru3B—P3B	2.3191 (10)
P1A—C14A	1.825 (4)	P1B—C14B	1.821 (4)
P1A—C8A	1.832 (4)	P1B—C8B	1.825 (4)
P1A—C1A	1.859 (4)	P1B—C1B	1.867 (4)
P2A—C35A	1.815 (4)	P2B—C35B	1.823 (4)
P2A—C29A	1.843 (4)	P2B—C29B	1.825 (4)
P2A—C41A	1.850 (4)	P2B—C41B	1.845 (4)
P3A—C42A	1.828 (4)	P3B—C42B	1.820 (4)
P3A—C48A	1.837 (4)	P3B—C48B	1.841 (4)
P3A—C41A	1.854 (4)	P3B—C41B	1.850 (4)
O1A—C20A	1.150 (5)	O1B—C20B	1.145 (4)
O2A—C21A	1.149 (4)	O2B—C21B	1.153 (5)
O3A—C22A	1.141 (5)	O3B—C22B	1.136 (5)
O4A—C23A	1.147 (5)	O4B—C23B	1.134 (5)
O5A—C24A	1.145 (5)	O5B—C24B	1.134 (5)
O6A—C25A	1.150 (5)	O6B—C25B	1.147 (5)
O7A—C26A	1.140 (5)	O7N—C26B	1.148 (5)
O8A—C27A	1.144 (5)	O8B—C27B	1.149 (5)
O9A—C28A	1.138 (5)	O9B—C28B	1.141 (5)
C1A—C2A	1.520 (5)	C1B—C2B	1.502 (5)
C1A—H1AA	0.9900	C1B—H1BA	0.9900
C1A—H1AB	0.9900	C1B—H1BB	0.9900
C2A—C3A	1.385 (5)	C2B—C3B	1.393 (6)
C2A—C7A	1.397 (5)	C2B—C7B	1.397 (5)
C3A—C4A	1.392 (6)	C3B—C4B	1.382 (6)
C3A—H3AA	0.9500	C3B—H3BA	0.9500
C4A—C5A	1.383 (6)	C4B—C5B	1.389 (6)
C4A—H4AA	0.9500	C4B—H4BA	0.9500
C5A—C6A	1.385 (6)	C5B—C6B	1.376 (6)
C5A—H5AA	0.9500	C5B—H5BA	0.9500
C6A—C7A	1.382 (6)	C6B—C7B	1.387 (6)
C6A—H6AA	0.9500	C6B—H6BA	0.9500
C7A—H7AA	0.9500	C7B—H7BA	0.9500
C8A—C9A	1.388 (5)	C8B—C9B	1.399 (5)
C8A—C13A	1.400 (5)	C8B—C13B	1.403 (5)
C9A—C10A	1.389 (5)	C9B—C10B	1.388 (5)
C9A—H9AA	0.9500	C9B—H9BA	0.9500
C10A—C11A	1.376 (6)	C10B—C11B	1.390 (5)
C10A—H10A	0.9500	C10B—H10B	0.9500
C11A—C12A	1.389 (6)	C11B—C12B	1.394 (6)
C11A—H11A	0.9500	C11B—H11B	0.9500
C12A—C13A	1.386 (5)	C12B—C13B	1.382 (5)
C12A—H12A	0.9500	C12B—H12B	0.9500
C13A—H13A	0.9500	C13B—H13B	0.9500
C14A—C19A	1.393 (5)	C14B—C19B	1.391 (6)
C14A—C15A	1.394 (6)	C14B—C15B	1.399 (5)
C15A—C16A	1.391 (5)	C15B—C16B	1.388 (5)
C15A—H15A	0.9500	C15B—H15B	0.9500
C16A—C17A	1.386 (6)	C16B—C17B	1.386 (6)
C16A—H16A	0.9500	C16B—H16B	0.9500
C17A—C18A	1.386 (6)	C17B—C18B	1.383 (6)
C17A—H17A	0.9500	C17B—H17B	0.9500
C18A—C19A	1.387 (5)	C18B—C19B	1.399 (6)
C18A—H18A	0.9500	C18B—H18B	0.9500
C19A—H19A	0.9500	C19B—H19B	0.9500
C29A—C30A	1.391 (5)	C29B—C30B	1.385 (6)
C29A—C34A	1.397 (6)	C29B—C34B	1.405 (5)
C30A—C31A	1.386 (5)	C30B—C31B	1.395 (6)
C30A—H30A	0.9500	C30B—H30B	0.9500
C31A—C32A	1.380 (6)	C31B—C32B	1.374 (6)
C31A—H31A	0.9500	C31B—H31B	0.9500
C32A—C33A	1.386 (6)	C32B—C33B	1.374 (6)
C32A—H32A	0.9500	C32B—H32B	0.9500
C33A—C34A	1.390 (5)	C33B—C34B	1.386 (5)
C33A—H33A	0.9500	C33B—H33B	0.9500
C34A—H34A	0.9500	C34B—H34B	0.9500
C35A—C36A	1.393 (5)	C35B—C36B	1.393 (5)
C35A—C40A	1.404 (5)	C35B—C40B	1.395 (5)
C36A—C37A	1.392 (6)	C36B—C37B	1.389 (6)
C36A—H36A	0.9500	C36B—H36B	0.9500
C37A—C38A	1.378 (6)	C37B—C38B	1.383 (6)
C37A—H37A	0.9500	C37B—H37B	0.9500
C38A—C39A	1.390 (6)	C38B—C39B	1.382 (6)
C38A—H38A	0.9500	C38B—H38B	0.9500
C39A—C40A	1.375 (6)	C39B—C40B	1.388 (5)
C39A—H39A	0.9500	C39B—H39B	0.9500
C40A—H40A	0.9500	C40B—H40B	0.9500
C41A—H41A	0.9900	C41B—H41C	0.9900
C41A—H41B	0.9900	C41B—H41D	0.9900
C42A—C43A	1.388 (6)	C42B—C43B	1.385 (5)
C42A—C47A	1.397 (5)	C42B—C47B	1.409 (5)
C43A—C44A	1.396 (5)	C43B—C44B	1.400 (6)
C43A—H43A	0.9500	C43B—H43B	0.9500
C44A—C45A	1.381 (6)	C44B—C45B	1.392 (6)
C44A—H44A	0.9500	C44B—H44B	0.9500
C45A—C46A	1.386 (6)	C45B—C46B	1.388 (6)
C45A—H45A	0.9500	C45B—H45B	0.9500
C46A—C47A	1.389 (6)	C46B—C47B	1.382 (6)
C46A—H46A	0.9500	C46B—H46B	0.9500
C47A—H47A	0.9500	C47B—H47B	0.9500
C48A—C49A	1.391 (5)	C48B—C53B	1.387 (5)
C48A—C53A	1.393 (6)	C48B—C49B	1.396 (5)
C49A—C50A	1.383 (6)	C49B—C50B	1.389 (6)
C49A—H49A	0.9500	C49B—H49B	0.9500
C50A—C51A	1.394 (7)	C50B—C51B	1.385 (6)
C50A—H50A	0.9500	C50B—H50B	0.9500
C51A—C52A	1.380 (6)	C51B—C52B	1.385 (6)
C51A—H51A	0.9500	C51B—H51B	0.9500
C52A—C53A	1.397 (5)	C52B—C53B	1.388 (5)
C52A—H52A	0.9500	C52B—H52B	0.9500
C53A—H53A	0.9500	C53B—H53B	0.9500
			
C21A—Ru1A—C20A	92.48 (16)	C21B—Ru1B—C20B	89.79 (15)
C21A—Ru1A—C22A	88.00 (16)	C21B—Ru1B—C22B	90.29 (16)
C20A—Ru1A—C22A	174.93 (16)	C20B—Ru1B—C22B	176.63 (15)
C21A—Ru1A—P1A	96.65 (12)	C21B—Ru1B—P1B	100.04 (12)
C20A—Ru1A—P1A	94.10 (11)	C20B—Ru1B—P1B	92.70 (11)
C22A—Ru1A—P1A	90.85 (11)	C22B—Ru1B—P1B	90.60 (11)
C21A—Ru1A—Ru3A	97.54 (11)	C21B—Ru1B—Ru2B	96.46 (12)
C20A—Ru1A—Ru3A	89.31 (11)	C20B—Ru1B—Ru2B	92.88 (10)
C22A—Ru1A—Ru3A	85.63 (11)	C22B—Ru1B—Ru2B	83.77 (11)
P1A—Ru1A—Ru3A	165.24 (3)	P1B—Ru1B—Ru2B	162.58 (3)
C21A—Ru1A—Ru2A	155.76 (12)	C21B—Ru1B—Ru3B	154.94 (12)
C20A—Ru1A—Ru2A	81.74 (11)	C20B—Ru1B—Ru3B	85.51 (11)
C22A—Ru1A—Ru2A	95.78 (11)	C22B—Ru1B—Ru3B	93.01 (11)
P1A—Ru1A—Ru2A	107.20 (3)	P1B—Ru1B—Ru3B	104.75 (3)
Ru3A—Ru1A—Ru2A	59.067 (10)	Ru2B—Ru1B—Ru3B	59.310 (10)
C24A—Ru2A—C25A	88.49 (16)	C24B—Ru2B—C25B	92.16 (16)
C24A—Ru2A—C23A	91.20 (17)	C24B—Ru2B—C23B	92.57 (17)
C25A—Ru2A—C23A	171.90 (17)	C25B—Ru2B—C23B	175.23 (16)
C24A—Ru2A—P2A	100.14 (12)	C24B—Ru2B—P2B	105.96 (12)
C25A—Ru2A—P2A	94.67 (12)	C25B—Ru2B—P2B	91.13 (12)
C23A—Ru2A—P2A	93.35 (13)	C23B—Ru2B—P2B	88.18 (12)
C24A—Ru2A—Ru3A	169.64 (12)	C24B—Ru2B—Ru3B	156.83 (11)
C25A—Ru2A—Ru3A	96.22 (11)	C25B—Ru2B—Ru3B	81.21 (11)
C23A—Ru2A—Ru3A	82.83 (11)	C23B—Ru2B—Ru3B	94.17 (12)
P2A—Ru2A—Ru3A	88.70 (3)	P2B—Ru2B—Ru3B	96.40 (3)
C24A—Ru2A—Ru1A	112.89 (12)	C24B—Ru2B—Ru1B	97.34 (12)
C25A—Ru2A—Ru1A	78.57 (11)	C25B—Ru2B—Ru1B	93.23 (11)
C23A—Ru2A—Ru1A	94.12 (12)	C23B—Ru2B—Ru1B	85.54 (11)
P2A—Ru2A—Ru1A	145.91 (3)	P2B—Ru2B—Ru1B	156.11 (3)
Ru3A—Ru2A—Ru1A	59.411 (10)	Ru3B—Ru2B—Ru1B	61.198 (10)
C27A—Ru3A—C28A	93.00 (17)	C27B—Ru3B—C26B	92.62 (17)
C27A—Ru3A—C26A	92.66 (17)	C27B—Ru3B—C28B	87.53 (16)
C28A—Ru3A—C26A	174.30 (16)	C26B—Ru3B—C28B	171.69 (16)
C27A—Ru3A—P3A	106.25 (12)	C27B—Ru3B—P3B	99.65 (12)
C28A—Ru3A—P3A	90.55 (12)	C26B—Ru3B—P3B	93.26 (12)
C26A—Ru3A—P3A	87.18 (12)	C28B—Ru3B—P3B	94.90 (11)
C27A—Ru3A—Ru2A	156.38 (12)	C27B—Ru3B—Ru2B	170.95 (12)
C28A—Ru3A—Ru2A	80.85 (11)	C26B—Ru3B—Ru2B	83.90 (12)
C26A—Ru3A—Ru2A	94.21 (11)	C28B—Ru3B—Ru2B	94.70 (11)
P3A—Ru3A—Ru2A	96.65 (3)	P3B—Ru3B—Ru2B	88.92 (3)
C27A—Ru3A—Ru1A	95.98 (12)	C27B—Ru3B—Ru1B	112.31 (12)
C28A—Ru3A—Ru1A	90.47 (11)	C26B—Ru3B—Ru1B	90.30 (11)
C26A—Ru3A—Ru1A	89.65 (12)	C28B—Ru3B—Ru1B	81.97 (11)
P3A—Ru3A—Ru1A	157.65 (3)	P3B—Ru3B—Ru1B	147.64 (3)
Ru2A—Ru3A—Ru1A	61.522 (10)	Ru2B—Ru3B—Ru1B	59.492 (10)
C14A—P1A—C8A	101.86 (17)	C14B—P1B—C8B	103.22 (17)
C14A—P1A—C1A	102.16 (18)	C14B—P1B—C1B	102.44 (18)
C8A—P1A—C1A	100.24 (17)	C8B—P1B—C1B	100.09 (18)
C14A—P1A—Ru1A	112.63 (12)	C14B—P1B—Ru1B	112.94 (12)
C8A—P1A—Ru1A	122.60 (13)	C8B—P1B—Ru1B	121.04 (13)
C1A—P1A—Ru1A	114.63 (12)	C1B—P1B—Ru1B	114.71 (12)
C35A—P2A—C29A	101.65 (17)	C35B—P2B—C29B	98.54 (17)
C35A—P2A—C41A	105.59 (18)	C35B—P2B—C41B	105.67 (17)
C29A—P2A—C41A	98.81 (16)	C29B—P2B—C41B	102.85 (17)
C35A—P2A—Ru2A	117.11 (13)	C35B—P2B—Ru2B	120.05 (13)
C29A—P2A—Ru2A	116.51 (13)	C29B—P2B—Ru2B	115.70 (13)
C41A—P2A—Ru2A	114.77 (12)	C41B—P2B—Ru2B	111.91 (12)
C42A—P3A—C48A	99.72 (17)	C42B—P3B—C48B	101.87 (17)
C42A—P3A—C41A	106.04 (18)	C42B—P3B—C41B	106.45 (17)
C48A—P3A—C41A	102.82 (17)	C48B—P3B—C41B	98.07 (16)
C42A—P3A—Ru3A	118.19 (13)	C42B—P3B—Ru3B	116.88 (13)
C48A—P3A—Ru3A	116.05 (13)	C48B—P3B—Ru3B	117.15 (13)
C41A—P3A—Ru3A	112.18 (12)	C41B—P3B—Ru3B	114.07 (12)
C2A—C1A—P1A	115.3 (3)	C2B—C1B—P1B	113.4 (3)
C2A—C1A—H1AA	108.5	C2B—C1B—H1BA	108.9
P1A—C1A—H1AA	108.5	P1B—C1B—H1BA	108.9
C2A—C1A—H1AB	108.5	C2B—C1B—H1BB	108.9
P1A—C1A—H1AB	108.5	P1B—C1B—H1BB	108.9
H1AA—C1A—H1AB	107.5	H1BA—C1B—H1BB	107.7
C3A—C2A—C7A	118.4 (4)	C3B—C2B—C7B	117.6 (4)
C3A—C2A—C1A	121.4 (4)	C3B—C2B—C1B	121.7 (3)
C7A—C2A—C1A	120.2 (3)	C7B—C2B—C1B	120.7 (4)
C2A—C3A—C4A	120.8 (4)	C4B—C3B—C2B	120.9 (4)
C2A—C3A—H3AA	119.6	C4B—C3B—H3BA	119.6
C4A—C3A—H3AA	119.6	C2B—C3B—H3BA	119.6
C5A—C4A—C3A	119.8 (4)	C3B—C4B—C5B	120.7 (4)
C5A—C4A—H4AA	120.1	C3B—C4B—H4BA	119.7
C3A—C4A—H4AA	120.1	C5B—C4B—H4BA	119.7
C4A—C5A—C6A	120.0 (4)	C6B—C5B—C4B	119.1 (4)
C4A—C5A—H5AA	120.0	C6B—C5B—H5BA	120.4
C6A—C5A—H5AA	120.0	C4B—C5B—H5BA	120.4
C7A—C6A—C5A	119.8 (4)	C5B—C6B—C7B	120.3 (4)
C7A—C6A—H6AA	120.1	C5B—C6B—H6BA	119.9
C5A—C6A—H6AA	120.1	C7B—C6B—H6BA	119.9
C6A—C7A—C2A	121.1 (4)	C6B—C7B—C2B	121.3 (4)
C6A—C7A—H7AA	119.5	C6B—C7B—H7BA	119.4
C2A—C7A—H7AA	119.5	C2B—C7B—H7BA	119.4
C9A—C8A—C13A	119.1 (4)	C9B—C8B—C13B	118.3 (3)
C9A—C8A—P1A	120.8 (3)	C9B—C8B—P1B	120.3 (3)
C13A—C8A—P1A	119.9 (3)	C13B—C8B—P1B	121.0 (3)
C8A—C9A—C10A	120.4 (4)	C10B—C9B—C8B	121.1 (4)
C8A—C9A—H9AA	119.8	C10B—C9B—H9BA	119.4
C10A—C9A—H9AA	119.8	C8B—C9B—H9BA	119.4
C11A—C10A—C9A	120.2 (4)	C9B—C10B—C11B	119.7 (4)
C11A—C10A—H10A	119.9	C9B—C10B—H10B	120.2
C9A—C10A—H10A	119.9	C11B—C10B—H10B	120.2
C10A—C11A—C12A	120.0 (4)	C10B—C11B—C12B	120.0 (4)
C10A—C11A—H11A	120.0	C10B—C11B—H11B	120.0
C12A—C11A—H11A	120.0	C12B—C11B—H11B	120.0
C13A—C12A—C11A	120.0 (4)	C13B—C12B—C11B	120.0 (4)
C13A—C12A—H12A	120.0	C13B—C12B—H12B	120.0
C11A—C12A—H12A	120.0	C11B—C12B—H12B	120.0
C12A—C13A—C8A	120.2 (4)	C12B—C13B—C8B	120.9 (4)
C12A—C13A—H13A	119.9	C12B—C13B—H13B	119.6
C8A—C13A—H13A	119.9	C8B—C13B—H13B	119.6
C19A—C14A—C15A	118.4 (4)	C19B—C14B—C15B	118.4 (4)
C19A—C14A—P1A	120.1 (3)	C19B—C14B—P1B	122.0 (3)
C15A—C14A—P1A	121.4 (3)	C15B—C14B—P1B	119.3 (3)
C16A—C15A—C14A	120.8 (4)	C16B—C15B—C14B	121.1 (4)
C16A—C15A—H15A	119.6	C16B—C15B—H15B	119.5
C14A—C15A—H15A	119.6	C14B—C15B—H15B	119.5
C17A—C16A—C15A	119.9 (4)	C17B—C16B—C15B	119.9 (4)
C17A—C16A—H16A	120.0	C17B—C16B—H16B	120.0
C15A—C16A—H16A	120.0	C15B—C16B—H16B	120.0
C16A—C17A—C18A	120.1 (4)	C18B—C17B—C16B	119.8 (4)
C16A—C17A—H17A	120.0	C18B—C17B—H17B	120.1
C18A—C17A—H17A	120.0	C16B—C17B—H17B	120.1
C17A—C18A—C19A	119.7 (4)	C17B—C18B—C19B	120.2 (4)
C17A—C18A—H18A	120.2	C17B—C18B—H18B	119.9
C19A—C18A—H18A	120.2	C19B—C18B—H18B	119.9
C18A—C19A—C14A	121.2 (4)	C14B—C19B—C18B	120.5 (4)
C18A—C19A—H19A	119.4	C14B—C19B—H19B	119.7
C14A—C19A—H19A	119.4	C18B—C19B—H19B	119.7
O1A—C20A—Ru1A	173.2 (3)	O1B—C20B—Ru1B	173.4 (3)
O2A—C21A—Ru1A	179.5 (4)	O2B—C21B—Ru1B	177.3 (4)
O3A—C22A—Ru1A	172.5 (3)	O3B—C22B—Ru1B	173.3 (3)
O4A—C23A—Ru2A	173.4 (3)	O4B—C23B—Ru2B	173.9 (3)
O5A—C24A—Ru2A	177.1 (4)	O5B—C24B—Ru2B	176.6 (3)
O6A—C25A—Ru2A	171.3 (3)	O6B—C25B—Ru2B	173.1 (3)
O7A—C26A—Ru3A	174.3 (3)	O7N—C26B—Ru3B	173.6 (3)
O8A—C27A—Ru3A	176.1 (4)	O8B—C27B—Ru3B	176.6 (4)
O9A—C28A—Ru3A	173.8 (3)	O9B—C28B—Ru3B	171.4 (3)
C30A—C29A—C34A	118.4 (4)	C30B—C29B—C34B	118.3 (3)
C30A—C29A—P2A	122.4 (3)	C30B—C29B—P2B	125.2 (3)
C34A—C29A—P2A	119.2 (3)	C34B—C29B—P2B	116.4 (3)
C31A—C30A—C29A	120.7 (4)	C29B—C30B—C31B	120.6 (4)
C31A—C30A—H30A	119.7	C29B—C30B—H30B	119.7
C29A—C30A—H30A	119.7	C31B—C30B—H30B	119.7
C32A—C31A—C30A	120.7 (4)	C32B—C31B—C30B	120.2 (4)
C32A—C31A—H31A	119.7	C32B—C31B—H31B	119.9
C30A—C31A—H31A	119.7	C30B—C31B—H31B	119.9
C31A—C32A—C33A	119.4 (4)	C33B—C32B—C31B	120.1 (4)
C31A—C32A—H32A	120.3	C33B—C32B—H32B	120.0
C33A—C32A—H32A	120.3	C31B—C32B—H32B	120.0
C32A—C33A—C34A	120.2 (4)	C32B—C33B—C34B	120.3 (4)
C32A—C33A—H33A	119.9	C32B—C33B—H33B	119.8
C34A—C33A—H33A	119.9	C34B—C33B—H33B	119.8
C33A—C34A—C29A	120.7 (4)	C33B—C34B—C29B	120.4 (4)
C33A—C34A—H34A	119.7	C33B—C34B—H34B	119.8
C29A—C34A—H34A	119.7	C29B—C34B—H34B	119.8
C36A—C35A—C40A	117.8 (4)	C36B—C35B—C40B	118.7 (4)
C36A—C35A—P2A	123.9 (3)	C36B—C35B—P2B	119.8 (3)
C40A—C35A—P2A	118.2 (3)	C40B—C35B—P2B	121.4 (3)
C37A—C36A—C35A	120.6 (4)	C37B—C36B—C35B	120.6 (4)
C37A—C36A—H36A	119.7	C37B—C36B—H36B	119.7
C35A—C36A—H36A	119.7	C35B—C36B—H36B	119.7
C38A—C37A—C36A	120.4 (4)	C38B—C37B—C36B	120.0 (4)
C38A—C37A—H37A	119.8	C38B—C37B—H37B	120.0
C36A—C37A—H37A	119.8	C36B—C37B—H37B	120.0
C37A—C38A—C39A	119.7 (4)	C39B—C38B—C37B	120.0 (4)
C37A—C38A—H38A	120.1	C39B—C38B—H38B	120.0
C39A—C38A—H38A	120.1	C37B—C38B—H38B	120.0
C40A—C39A—C38A	119.9 (4)	C38B—C39B—C40B	120.1 (4)
C40A—C39A—H39A	120.1	C38B—C39B—H39B	119.9
C38A—C39A—H39A	120.1	C40B—C39B—H39B	119.9
C39A—C40A—C35A	121.4 (4)	C39B—C40B—C35B	120.6 (4)
C39A—C40A—H40A	119.3	C39B—C40B—H40B	119.7
C35A—C40A—H40A	119.3	C35B—C40B—H40B	119.7
P2A—C41A—P3A	115.5 (2)	P2B—C41B—P3B	115.6 (2)
P2A—C41A—H41A	108.4	P2B—C41B—H41C	108.4
P3A—C41A—H41A	108.4	P3B—C41B—H41C	108.4
P2A—C41A—H41B	108.4	P2B—C41B—H41D	108.4
P3A—C41A—H41B	108.4	P3B—C41B—H41D	108.4
H41A—C41A—H41B	107.5	H41C—C41B—H41D	107.4
C43A—C42A—C47A	118.9 (4)	C43B—C42B—C47B	118.7 (4)
C43A—C42A—P3A	121.2 (3)	C43B—C42B—P3B	123.9 (3)
C47A—C42A—P3A	119.8 (3)	C47B—C42B—P3B	117.5 (3)
C42A—C43A—C44A	120.7 (4)	C42B—C43B—C44B	120.7 (4)
C42A—C43A—H43A	119.6	C42B—C43B—H43B	119.6
C44A—C43A—H43A	119.6	C44B—C43B—H43B	119.6
C45A—C44A—C43A	119.8 (4)	C45B—C44B—C43B	119.9 (4)
C45A—C44A—H44A	120.1	C45B—C44B—H44B	120.0
C43A—C44A—H44A	120.1	C43B—C44B—H44B	120.0
C44A—C45A—C46A	120.0 (4)	C46B—C45B—C44B	119.6 (4)
C44A—C45A—H45A	120.0	C46B—C45B—H45B	120.2
C46A—C45A—H45A	120.0	C44B—C45B—H45B	120.2
C45A—C46A—C47A	120.2 (4)	C47B—C46B—C45B	120.5 (4)
C45A—C46A—H46A	119.9	C47B—C46B—H46B	119.8
C47A—C46A—H46A	119.9	C45B—C46B—H46B	119.8
C46A—C47A—C42A	120.3 (4)	C46B—C47B—C42B	120.6 (4)
C46A—C47A—H47A	119.8	C46B—C47B—H47B	119.7
C42A—C47A—H47A	119.8	C42B—C47B—H47B	119.7
C49A—C48A—C53A	118.7 (4)	C53B—C48B—C49B	118.4 (4)
C49A—C48A—P3A	116.8 (3)	C53B—C48B—P3B	119.8 (3)
C53A—C48A—P3A	124.5 (3)	C49B—C48B—P3B	121.7 (3)
C50A—C49A—C48A	121.1 (4)	C50B—C49B—C48B	120.5 (4)
C50A—C49A—H49A	119.4	C50B—C49B—H49B	119.8
C48A—C49A—H49A	119.4	C48B—C49B—H49B	119.8
C49A—C50A—C51A	120.0 (4)	C51B—C50B—C49B	120.2 (4)
C49A—C50A—H50A	120.0	C51B—C50B—H50B	119.9
C51A—C50A—H50A	120.0	C49B—C50B—H50B	119.9
C52A—C51A—C50A	119.4 (4)	C52B—C51B—C50B	120.0 (4)
C52A—C51A—H51A	120.3	C52B—C51B—H51B	120.0
C50A—C51A—H51A	120.3	C50B—C51B—H51B	120.0
C51A—C52A—C53A	120.5 (4)	C51B—C52B—C53B	119.5 (4)
C51A—C52A—H52A	119.7	C51B—C52B—H52B	120.3
C53A—C52A—H52A	119.7	C53B—C52B—H52B	120.3
C48A—C53A—C52A	120.2 (4)	C48B—C53B—C52B	121.4 (4)
C48A—C53A—H53A	119.9	C48B—C53B—H53B	119.3
C52A—C53A—H53A	119.9	C52B—C53B—H53B	119.3
			
C21A—Ru1A—Ru2A—C24A	−155.6 (3)	C50A—C51A—C52A—C53A	1.8 (7)
C20A—Ru1A—Ru2A—C24A	−78.05 (17)	C49A—C48A—C53A—C52A	0.0 (6)
C22A—Ru1A—Ru2A—C24A	106.41 (17)	P3A—C48A—C53A—C52A	178.6 (3)
P1A—Ru1A—Ru2A—C24A	13.71 (13)	C51A—C52A—C53A—C48A	−1.0 (7)
Ru3A—Ru1A—Ru2A—C24A	−172.22 (13)	C21B—Ru1B—Ru2B—C24B	16.11 (17)
C21A—Ru1A—Ru2A—C25A	121.0 (3)	C20B—Ru1B—Ru2B—C24B	106.22 (16)
C20A—Ru1A—Ru2A—C25A	−161.48 (15)	C22B—Ru1B—Ru2B—C24B	−73.48 (16)
C22A—Ru1A—Ru2A—C25A	22.98 (16)	P1B—Ru1B—Ru2B—C24B	−145.24 (15)
P1A—Ru1A—Ru2A—C25A	−69.71 (12)	Ru3B—Ru1B—Ru2B—C24B	−170.73 (12)
Ru3A—Ru1A—Ru2A—C25A	104.35 (11)	C21B—Ru1B—Ru2B—C25B	108.70 (16)
C21A—Ru1A—Ru2A—C23A	−62.5 (3)	C20B—Ru1B—Ru2B—C25B	−161.18 (15)
C20A—Ru1A—Ru2A—C23A	15.00 (16)	C22B—Ru1B—Ru2B—C25B	19.12 (16)
C22A—Ru1A—Ru2A—C23A	−160.55 (16)	P1B—Ru1B—Ru2B—C25B	−52.64 (15)
P1A—Ru1A—Ru2A—C23A	106.76 (12)	Ru3B—Ru1B—Ru2B—C25B	−78.13 (11)
Ru3A—Ru1A—Ru2A—C23A	−79.17 (12)	C21B—Ru1B—Ru2B—C23B	−75.91 (17)
C21A—Ru1A—Ru2A—P2A	39.7 (3)	C20B—Ru1B—Ru2B—C23B	14.20 (16)
C20A—Ru1A—Ru2A—P2A	117.18 (12)	C22B—Ru1B—Ru2B—C23B	−165.50 (16)
C22A—Ru1A—Ru2A—P2A	−58.36 (12)	P1B—Ru1B—Ru2B—C23B	122.74 (15)
P1A—Ru1A—Ru2A—P2A	−151.05 (5)	Ru3B—Ru1B—Ru2B—C23B	97.25 (12)
Ru3A—Ru1A—Ru2A—P2A	23.01 (5)	C21B—Ru1B—Ru2B—P2B	−151.15 (13)
C21A—Ru1A—Ru2A—Ru3A	16.6 (3)	C20B—Ru1B—Ru2B—P2B	−61.04 (12)
C20A—Ru1A—Ru2A—Ru3A	94.17 (11)	C22B—Ru1B—Ru2B—P2B	119.26 (13)
C22A—Ru1A—Ru2A—Ru3A	−81.37 (11)	P1B—Ru1B—Ru2B—P2B	47.50 (12)
P1A—Ru1A—Ru2A—Ru3A	−174.06 (3)	Ru3B—Ru1B—Ru2B—P2B	22.01 (7)
C24A—Ru2A—Ru3A—C27A	63.1 (7)	C21B—Ru1B—Ru2B—Ru3B	−173.16 (11)
C25A—Ru2A—Ru3A—C27A	−53.6 (3)	C20B—Ru1B—Ru2B—Ru3B	−83.04 (10)
C23A—Ru2A—Ru3A—C27A	118.3 (3)	C22B—Ru1B—Ru2B—Ru3B	97.25 (11)
P2A—Ru2A—Ru3A—C27A	−148.1 (3)	P1B—Ru1B—Ru2B—Ru3B	25.50 (9)
Ru1A—Ru2A—Ru3A—C27A	19.2 (3)	C24B—Ru2B—Ru3B—C26B	117.9 (3)
C24A—Ru2A—Ru3A—C28A	139.4 (7)	C25B—Ru2B—Ru3B—C26B	−167.41 (16)
C25A—Ru2A—Ru3A—C28A	22.77 (16)	C23B—Ru2B—Ru3B—C26B	11.38 (17)
C23A—Ru2A—Ru3A—C28A	−165.33 (17)	P2B—Ru2B—Ru3B—C26B	−77.26 (12)
P2A—Ru2A—Ru3A—C28A	−71.78 (12)	Ru1B—Ru2B—Ru3B—C26B	93.96 (12)
Ru1A—Ru2A—Ru3A—C28A	95.56 (12)	C24B—Ru2B—Ru3B—C28B	−53.9 (3)
C24A—Ru2A—Ru3A—C26A	−43.4 (7)	C25B—Ru2B—Ru3B—C28B	20.82 (16)
C25A—Ru2A—Ru3A—C26A	−160.09 (17)	C23B—Ru2B—Ru3B—C28B	−160.39 (16)
C23A—Ru2A—Ru3A—C26A	11.80 (17)	P2B—Ru2B—Ru3B—C28B	110.97 (11)
P2A—Ru2A—Ru3A—C26A	105.35 (12)	Ru1B—Ru2B—Ru3B—C28B	−77.81 (11)
Ru1A—Ru2A—Ru3A—C26A	−87.31 (12)	C24B—Ru2B—Ru3B—P3B	−148.7 (3)
C24A—Ru2A—Ru3A—P3A	−131.1 (7)	C25B—Ru2B—Ru3B—P3B	−74.01 (12)
C25A—Ru2A—Ru3A—P3A	112.26 (12)	C23B—Ru2B—Ru3B—P3B	104.78 (12)
C23A—Ru2A—Ru3A—P3A	−75.84 (13)	P2B—Ru2B—Ru3B—P3B	16.14 (4)
P2A—Ru2A—Ru3A—P3A	17.70 (4)	Ru1B—Ru2B—Ru3B—P3B	−172.64 (3)
Ru1A—Ru2A—Ru3A—P3A	−174.96 (3)	C24B—Ru2B—Ru3B—Ru1B	24.0 (3)
C24A—Ru2A—Ru3A—Ru1A	43.9 (7)	C25B—Ru2B—Ru3B—Ru1B	98.63 (12)
C25A—Ru2A—Ru3A—Ru1A	−72.78 (12)	C23B—Ru2B—Ru3B—Ru1B	−82.58 (12)
C23A—Ru2A—Ru3A—Ru1A	99.11 (12)	P2B—Ru2B—Ru3B—Ru1B	−171.22 (3)
P2A—Ru2A—Ru3A—Ru1A	−167.34 (3)	C21B—Ru1B—Ru3B—C27B	−159.5 (3)
C21A—Ru1A—Ru3A—C27A	14.43 (17)	C20B—Ru1B—Ru3B—C27B	−79.65 (17)
C20A—Ru1A—Ru3A—C27A	106.84 (16)	C22B—Ru1B—Ru3B—C27B	103.37 (17)
C22A—Ru1A—Ru3A—C27A	−72.97 (17)	P1B—Ru1B—Ru3B—C27B	11.96 (13)
P1A—Ru1A—Ru3A—C27A	−149.57 (16)	Ru2B—Ru1B—Ru3B—C27B	−175.70 (13)
Ru2A—Ru1A—Ru3A—C27A	−172.38 (12)	C21B—Ru1B—Ru3B—C26B	−66.5 (3)
C21A—Ru1A—Ru3A—C28A	107.50 (16)	C20B—Ru1B—Ru3B—C26B	13.31 (15)
C20A—Ru1A—Ru3A—C28A	−160.09 (15)	C22B—Ru1B—Ru3B—C26B	−163.67 (16)
C22A—Ru1A—Ru3A—C28A	20.10 (16)	P1B—Ru1B—Ru3B—C26B	104.92 (12)
P1A—Ru1A—Ru3A—C28A	−56.50 (16)	Ru2B—Ru1B—Ru3B—C26B	−82.74 (12)
Ru2A—Ru1A—Ru3A—C28A	−79.32 (11)	C21B—Ru1B—Ru3B—C28B	116.5 (3)
C21A—Ru1A—Ru3A—C26A	−78.21 (16)	C20B—Ru1B—Ru3B—C28B	−163.63 (15)
C20A—Ru1A—Ru3A—C26A	14.20 (15)	C22B—Ru1B—Ru3B—C28B	19.39 (15)
C22A—Ru1A—Ru3A—C26A	−165.60 (16)	P1B—Ru1B—Ru3B—C28B	−72.02 (11)
P1A—Ru1A—Ru3A—C26A	117.80 (16)	Ru2B—Ru1B—Ru3B—C28B	100.32 (11)
Ru2A—Ru1A—Ru3A—C26A	94.98 (11)	C21B—Ru1B—Ru3B—P3B	30.1 (3)
C21A—Ru1A—Ru3A—P3A	−159.91 (14)	C20B—Ru1B—Ru3B—P3B	109.89 (11)
C20A—Ru1A—Ru3A—P3A	−67.50 (13)	C22B—Ru1B—Ru3B—P3B	−67.09 (12)
C22A—Ru1A—Ru3A—P3A	112.70 (13)	P1B—Ru1B—Ru3B—P3B	−158.50 (6)
P1A—Ru1A—Ru3A—P3A	36.10 (14)	Ru2B—Ru1B—Ru3B—P3B	13.84 (5)
Ru2A—Ru1A—Ru3A—P3A	13.28 (7)	C21B—Ru1B—Ru3B—Ru2B	16.2 (3)
C21A—Ru1A—Ru3A—Ru2A	−173.19 (12)	C20B—Ru1B—Ru3B—Ru2B	96.04 (10)
C20A—Ru1A—Ru3A—Ru2A	−80.78 (11)	C22B—Ru1B—Ru3B—Ru2B	−80.93 (11)
C22A—Ru1A—Ru3A—Ru2A	99.42 (11)	P1B—Ru1B—Ru3B—Ru2B	−172.34 (3)
P1A—Ru1A—Ru3A—Ru2A	22.82 (11)	C21B—Ru1B—P1B—C14B	−94.96 (18)
C21A—Ru1A—P1A—C14A	−93.36 (18)	C20B—Ru1B—P1B—C14B	174.77 (18)
C20A—Ru1A—P1A—C14A	173.65 (18)	C22B—Ru1B—P1B—C14B	−4.56 (18)
C22A—Ru1A—P1A—C14A	−5.28 (18)	Ru2B—Ru1B—P1B—C14B	66.21 (17)
Ru3A—Ru1A—P1A—C14A	70.66 (18)	Ru3B—Ru1B—P1B—C14B	88.71 (14)
Ru2A—Ru1A—P1A—C14A	91.04 (14)	C21B—Ru1B—P1B—C8B	142.05 (18)
C21A—Ru1A—P1A—C8A	144.57 (19)	C20B—Ru1B—P1B—C8B	51.78 (18)
C20A—Ru1A—P1A—C8A	51.58 (18)	C22B—Ru1B—P1B—C8B	−127.55 (18)
C22A—Ru1A—P1A—C8A	−127.35 (19)	Ru2B—Ru1B—P1B—C8B	−56.79 (18)
Ru3A—Ru1A—P1A—C8A	−51.4 (2)	Ru3B—Ru1B—P1B—C8B	−34.28 (15)
Ru2A—Ru1A—P1A—C8A	−31.03 (15)	C21B—Ru1B—P1B—C1B	21.92 (18)
C21A—Ru1A—P1A—C1A	22.88 (18)	C20B—Ru1B—P1B—C1B	−68.35 (18)
C20A—Ru1A—P1A—C1A	−70.10 (18)	C22B—Ru1B—P1B—C1B	112.32 (18)
C22A—Ru1A—P1A—C1A	110.96 (18)	Ru2B—Ru1B—P1B—C1B	−176.92 (15)
Ru3A—Ru1A—P1A—C1A	−173.10 (15)	Ru3B—Ru1B—P1B—C1B	−154.41 (14)
Ru2A—Ru1A—P1A—C1A	−152.72 (14)	C24B—Ru2B—P2B—C35B	50.18 (18)
C24A—Ru2A—P2A—C35A	−93.65 (18)	C25B—Ru2B—P2B—C35B	−42.39 (17)
C25A—Ru2A—P2A—C35A	−4.34 (18)	C23B—Ru2B—P2B—C35B	142.33 (18)
C23A—Ru2A—P2A—C35A	174.53 (18)	Ru3B—Ru2B—P2B—C35B	−123.68 (13)
Ru3A—Ru2A—P2A—C35A	91.79 (14)	Ru1B—Ru2B—P2B—C35B	−142.97 (13)
Ru1A—Ru2A—P2A—C35A	72.12 (15)	C24B—Ru2B—P2B—C29B	−67.78 (19)
C24A—Ru2A—P2A—C29A	26.94 (18)	C25B—Ru2B—P2B—C29B	−160.35 (18)
C25A—Ru2A—P2A—C29A	116.24 (17)	C23B—Ru2B—P2B—C29B	24.37 (18)
C23A—Ru2A—P2A—C29A	−64.88 (17)	Ru3B—Ru2B—P2B—C29B	118.37 (14)
Ru3A—Ru2A—P2A—C29A	−147.63 (13)	Ru1B—Ru2B—P2B—C29B	99.07 (15)
Ru1A—Ru2A—P2A—C29A	−167.30 (12)	C24B—Ru2B—P2B—C41B	174.86 (18)
C24A—Ru2A—P2A—C41A	141.69 (18)	C25B—Ru2B—P2B—C41B	82.29 (17)
C25A—Ru2A—P2A—C41A	−129.01 (18)	C23B—Ru2B—P2B—C41B	−92.99 (18)
C23A—Ru2A—P2A—C41A	49.87 (18)	Ru3B—Ru2B—P2B—C41B	1.00 (14)
Ru3A—Ru2A—P2A—C41A	−32.88 (14)	Ru1B—Ru2B—P2B—C41B	−18.29 (16)
Ru1A—Ru2A—P2A—C41A	−52.55 (15)	C27B—Ru3B—P3B—C42B	−90.63 (19)
C27A—Ru3A—P3A—C42A	48.17 (19)	C26B—Ru3B—P3B—C42B	176.15 (18)
C28A—Ru3A—P3A—C42A	−45.12 (17)	C28B—Ru3B—P3B—C42B	−2.30 (18)
C26A—Ru3A—P3A—C42A	140.12 (17)	Ru2B—Ru3B—P3B—C42B	92.32 (14)
Ru2A—Ru3A—P3A—C42A	−125.97 (13)	Ru1B—Ru3B—P3B—C42B	80.42 (15)
Ru1A—Ru3A—P3A—C42A	−137.70 (14)	C27B—Ru3B—P3B—C48B	30.65 (18)
C27A—Ru3A—P3A—C48A	−70.20 (19)	C26B—Ru3B—P3B—C48B	−62.58 (18)
C28A—Ru3A—P3A—C48A	−163.49 (18)	C28B—Ru3B—P3B—C48B	118.97 (17)
C26A—Ru3A—P3A—C48A	21.74 (18)	Ru2B—Ru3B—P3B—C48B	−146.40 (13)
Ru2A—Ru3A—P3A—C48A	115.66 (14)	Ru1B—Ru3B—P3B—C48B	−158.30 (13)
Ru1A—Ru3A—P3A—C48A	103.93 (15)	C27B—Ru3B—P3B—C41B	144.34 (18)
C27A—Ru3A—P3A—C41A	172.02 (18)	C26B—Ru3B—P3B—C41B	51.12 (18)
C28A—Ru3A—P3A—C41A	78.74 (18)	C28B—Ru3B—P3B—C41B	−127.33 (17)
C26A—Ru3A—P3A—C41A	−96.03 (18)	Ru2B—Ru3B—P3B—C41B	−32.71 (13)
Ru2A—Ru3A—P3A—C41A	−2.12 (14)	Ru1B—Ru3B—P3B—C41B	−44.61 (15)
Ru1A—Ru3A—P3A—C41A	−13.84 (17)	C14B—P1B—C1B—C2B	−173.1 (3)
C14A—P1A—C1A—C2A	−167.3 (3)	C8B—P1B—C1B—C2B	−67.0 (3)
C8A—P1A—C1A—C2A	−62.7 (3)	Ru1B—P1B—C1B—C2B	64.2 (3)
Ru1A—P1A—C1A—C2A	70.6 (3)	P1B—C1B—C2B—C3B	74.7 (4)
P1A—C1A—C2A—C3A	−97.4 (4)	P1B—C1B—C2B—C7B	−103.7 (4)
P1A—C1A—C2A—C7A	84.3 (4)	C7B—C2B—C3B—C4B	4.5 (6)
C7A—C2A—C3A—C4A	2.7 (6)	C1B—C2B—C3B—C4B	−173.9 (4)
C1A—C2A—C3A—C4A	−175.6 (4)	C2B—C3B—C4B—C5B	−3.6 (6)
C2A—C3A—C4A—C5A	−1.9 (6)	C3B—C4B—C5B—C6B	−0.4 (7)
C3A—C4A—C5A—C6A	0.5 (7)	C4B—C5B—C6B—C7B	3.2 (6)
C4A—C5A—C6A—C7A	0.1 (7)	C5B—C6B—C7B—C2B	−2.2 (6)
C5A—C6A—C7A—C2A	0.7 (7)	C3B—C2B—C7B—C6B	−1.7 (6)
C3A—C2A—C7A—C6A	−2.1 (6)	C1B—C2B—C7B—C6B	176.8 (4)
C1A—C2A—C7A—C6A	176.2 (4)	C14B—P1B—C8B—C9B	−151.7 (3)
C14A—P1A—C8A—C9A	−150.7 (3)	C1B—P1B—C8B—C9B	102.9 (3)
C1A—P1A—C8A—C9A	104.4 (3)	Ru1B—P1B—C8B—C9B	−24.2 (4)
Ru1A—P1A—C8A—C9A	−23.7 (4)	C14B—P1B—C8B—C13B	36.5 (4)
C14A—P1A—C8A—C13A	34.9 (4)	C1B—P1B—C8B—C13B	−69.0 (3)
C1A—P1A—C8A—C13A	−70.0 (3)	Ru1B—P1B—C8B—C13B	164.0 (3)
Ru1A—P1A—C8A—C13A	161.8 (3)	C13B—C8B—C9B—C10B	1.5 (6)
C13A—C8A—C9A—C10A	−0.7 (6)	P1B—C8B—C9B—C10B	−170.5 (3)
P1A—C8A—C9A—C10A	−175.2 (3)	C8B—C9B—C10B—C11B	−1.7 (6)
C8A—C9A—C10A—C11A	−0.6 (6)	C9B—C10B—C11B—C12B	0.0 (6)
C9A—C10A—C11A—C12A	1.2 (6)	C10B—C11B—C12B—C13B	1.8 (6)
C10A—C11A—C12A—C13A	−0.4 (6)	C11B—C12B—C13B—C8B	−2.0 (6)
C11A—C12A—C13A—C8A	−0.9 (6)	C9B—C8B—C13B—C12B	0.4 (6)
C9A—C8A—C13A—C12A	1.5 (6)	P1B—C8B—C13B—C12B	172.3 (3)
P1A—C8A—C13A—C12A	176.0 (3)	C8B—P1B—C14B—C19B	−137.6 (3)
C8A—P1A—C14A—C19A	48.6 (3)	C1B—P1B—C14B—C19B	−34.0 (4)
C1A—P1A—C14A—C19A	151.9 (3)	Ru1B—P1B—C14B—C19B	90.0 (3)
Ru1A—P1A—C14A—C19A	−84.6 (3)	C8B—P1B—C14B—C15B	48.7 (3)
C8A—P1A—C14A—C15A	−136.0 (3)	C1B—P1B—C14B—C15B	152.3 (3)
C1A—P1A—C14A—C15A	−32.6 (4)	Ru1B—P1B—C14B—C15B	−83.8 (3)
Ru1A—P1A—C14A—C15A	90.9 (3)	C19B—C14B—C15B—C16B	−0.2 (6)
C19A—C14A—C15A—C16A	−1.2 (6)	P1B—C14B—C15B—C16B	173.7 (3)
P1A—C14A—C15A—C16A	−176.7 (3)	C14B—C15B—C16B—C17B	0.0 (6)
C14A—C15A—C16A—C17A	1.0 (6)	C15B—C16B—C17B—C18B	0.5 (6)
C15A—C16A—C17A—C18A	−0.3 (6)	C16B—C17B—C18B—C19B	−0.7 (6)
C16A—C17A—C18A—C19A	−0.2 (6)	C15B—C14B—C19B—C18B	0.0 (6)
C17A—C18A—C19A—C14A	0.1 (6)	P1B—C14B—C19B—C18B	−173.8 (3)
C15A—C14A—C19A—C18A	0.6 (6)	C17B—C18B—C19B—C14B	0.5 (6)
P1A—C14A—C19A—C18A	176.2 (3)	Ru3B—Ru1B—C20B—O1B	179 (100)
C20A—Ru1A—C21A—O2A	169 (100)	C35B—P2B—C29B—C30B	99.8 (4)
Ru3A—Ru1A—C21A—O2A	−102 (100)	C41B—P2B—C29B—C30B	−8.6 (4)
Ru2A—Ru1A—C21A—O2A	−116 (100)	Ru2B—P2B—C29B—C30B	−130.9 (3)
Ru2A—Ru1A—C22A—O3A	−180 (100)	C35B—P2B—C29B—C34B	−75.8 (3)
C35A—P2A—C29A—C30A	−25.8 (3)	C41B—P2B—C29B—C34B	175.9 (3)
C41A—P2A—C29A—C30A	82.2 (3)	Ru2B—P2B—C29B—C34B	53.6 (3)
Ru2A—P2A—C29A—C30A	−154.3 (3)	C34B—C29B—C30B—C31B	−3.1 (6)
C35A—P2A—C29A—C34A	156.2 (3)	P2B—C29B—C30B—C31B	−178.5 (3)
C41A—P2A—C29A—C34A	−95.8 (3)	C29B—C30B—C31B—C32B	1.6 (7)
Ru2A—P2A—C29A—C34A	27.7 (3)	C30B—C31B—C32B—C33B	0.7 (7)
C34A—C29A—C30A—C31A	−0.5 (5)	C31B—C32B—C33B—C34B	−1.4 (7)
P2A—C29A—C30A—C31A	−178.5 (3)	C32B—C33B—C34B—C29B	−0.1 (6)
C29A—C30A—C31A—C32A	0.9 (6)	C30B—C29B—C34B—C33B	2.4 (6)
C30A—C31A—C32A—C33A	−0.8 (6)	P2B—C29B—C34B—C33B	178.2 (3)
C31A—C32A—C33A—C34A	0.1 (6)	C29B—P2B—C35B—C36B	−58.6 (3)
C32A—C33A—C34A—C29A	0.3 (6)	C41B—P2B—C35B—C36B	47.4 (3)
C30A—C29A—C34A—C33A	−0.2 (5)	Ru2B—P2B—C35B—C36B	175.0 (3)
P2A—C29A—C34A—C33A	177.9 (3)	C29B—P2B—C35B—C40B	116.7 (3)
C29A—P2A—C35A—C36A	115.1 (3)	C41B—P2B—C35B—C40B	−137.3 (3)
C41A—P2A—C35A—C36A	12.4 (4)	Ru2B—P2B—C35B—C40B	−9.7 (3)
Ru2A—P2A—C35A—C36A	−116.8 (3)	C40B—C35B—C36B—C37B	1.3 (6)
C29A—P2A—C35A—C40A	−64.3 (3)	P2B—C35B—C36B—C37B	176.7 (3)
C41A—P2A—C35A—C40A	−167.0 (3)	C35B—C36B—C37B—C38B	−0.6 (6)
Ru2A—P2A—C35A—C40A	63.9 (3)	C36B—C37B—C38B—C39B	−0.9 (6)
C40A—C35A—C36A—C37A	0.2 (6)	C37B—C38B—C39B—C40B	1.5 (6)
P2A—C35A—C36A—C37A	−179.1 (3)	C38B—C39B—C40B—C35B	−0.8 (6)
C35A—C36A—C37A—C38A	1.3 (6)	C36B—C35B—C40B—C39B	−0.6 (5)
C36A—C37A—C38A—C39A	−0.9 (7)	P2B—C35B—C40B—C39B	−176.0 (3)
C37A—C38A—C39A—C40A	−1.0 (6)	C35B—P2B—C41B—P3B	107.6 (2)
C38A—C39A—C40A—C35A	2.6 (6)	C29B—P2B—C41B—P3B	−149.6 (2)
C36A—C35A—C40A—C39A	−2.2 (6)	Ru2B—P2B—C41B—P3B	−24.7 (2)
P2A—C35A—C40A—C39A	177.2 (3)	C42B—P3B—C41B—P2B	−88.3 (2)
C35A—P2A—C41A—P3A	−90.8 (2)	C48B—P3B—C41B—P2B	166.7 (2)
C29A—P2A—C41A—P3A	164.4 (2)	Ru3B—P3B—C41B—P2B	42.1 (2)
Ru2A—P2A—C41A—P3A	39.7 (3)	C48B—P3B—C42B—C43B	115.4 (4)
C42A—P3A—C41A—P2A	109.5 (2)	C41B—P3B—C42B—C43B	13.2 (4)
C48A—P3A—C41A—P2A	−146.2 (2)	Ru3B—P3B—C42B—C43B	−115.6 (3)
Ru3A—P3A—C41A—P2A	−20.9 (3)	C48B—P3B—C42B—C47B	−65.3 (3)
C48A—P3A—C42A—C43A	119.5 (3)	C41B—P3B—C42B—C47B	−167.5 (3)
C41A—P3A—C42A—C43A	−134.1 (3)	Ru3B—P3B—C42B—C47B	63.7 (3)
Ru3A—P3A—C42A—C43A	−7.2 (4)	C47B—C42B—C43B—C44B	0.2 (6)
C48A—P3A—C42A—C47A	−56.0 (3)	P3B—C42B—C43B—C44B	179.5 (3)
C41A—P3A—C42A—C47A	50.5 (3)	C42B—C43B—C44B—C45B	1.1 (7)
Ru3A—P3A—C42A—C47A	177.3 (3)	C43B—C44B—C45B—C46B	−1.1 (7)
C47A—C42A—C43A—C44A	−1.3 (6)	C44B—C45B—C46B—C47B	−0.1 (7)
P3A—C42A—C43A—C44A	−176.8 (3)	C45B—C46B—C47B—C42B	1.4 (6)
C42A—C43A—C44A—C45A	−0.4 (6)	C43B—C42B—C47B—C46B	−1.4 (6)
C43A—C44A—C45A—C46A	1.7 (6)	P3B—C42B—C47B—C46B	179.2 (3)
C44A—C45A—C46A—C47A	−1.2 (6)	C42B—P3B—C48B—C53B	154.1 (3)
C45A—C46A—C47A—C42A	−0.5 (6)	C41B—P3B—C48B—C53B	−97.1 (3)
C43A—C42A—C47A—C46A	1.8 (6)	Ru3B—P3B—C48B—C53B	25.3 (3)
P3A—C42A—C47A—C46A	177.4 (3)	C42B—P3B—C48B—C49B	−30.6 (3)
C42A—P3A—C48A—C49A	−58.6 (3)	C41B—P3B—C48B—C49B	78.2 (3)
C41A—P3A—C48A—C49A	−167.6 (3)	Ru3B—P3B—C48B—C49B	−159.4 (3)
Ru3A—P3A—C48A—C49A	69.6 (3)	C53B—C48B—C49B—C50B	−1.5 (6)
C42A—P3A—C48A—C53A	122.8 (4)	P3B—C48B—C49B—C50B	−177.0 (3)
C41A—P3A—C48A—C53A	13.8 (4)	C48B—C49B—C50B—C51B	0.8 (6)
Ru3A—P3A—C48A—C53A	−109.0 (3)	C49B—C50B—C51B—C52B	0.2 (6)
C53A—C48A—C49A—C50A	0.3 (6)	C50B—C51B—C52B—C53B	−0.4 (6)
P3A—C48A—C49A—C50A	−178.4 (3)	C49B—C48B—C53B—C52B	1.4 (6)
C48A—C49A—C50A—C51A	0.5 (7)	P3B—C48B—C53B—C52B	176.8 (3)
C49A—C50A—C51A—C52A	−1.5 (7)	C51B—C52B—C53B—C48B	−0.4 (6)

### Hydrogen-bond geometry (Å, º)

Cg1 and Cg2 are the centroids of the C8B–C13B and C29A–C34A rings, respectively.

**Table:**

*D*—H···*A*	*D*—H	H···*A*	*D*···*A*	*D*—H···*A*
C5*A*—H5*AA*···O1*A*^i^	0.95	2.55	3.221 (5)	127
C10*B*—H10*B*···O1*B*^ii^	0.95	2.58	3.250 (5)	128
C3*B*—H3*BA*···*Cg*1	0.95	2.78	3.485 (4)	131
C12*B*—H12*B*···*Cg*2^iii^	0.95	2.67	3.539 (4)	153

Symmetry codes: (i) −*x*+1, −*y*, −*z*+1; (ii) −*x*+2, −*y*, −*z*; (iii) *x*, *y*, *z*−1.
